# *Arabidopsis thaliana* SPF1 and SPF2 are nuclear-located ULP2-like SUMO proteases that act downstream of SIZ1 in plant development

**DOI:** 10.1093/jxb/ery265

**Published:** 2018-07-25

**Authors:** Pedro Humberto Castro, Miguel Ângelo Santos, Sara Freitas, Pepe Cana-Quijada, Tiago Lourenço, Mafalda A A Rodrigues, Fátima Fonseca, Javier Ruiz-Albert, Jorge E Azevedo, Rui Manuel Tavares, Araceli G Castillo, Eduardo R Bejarano, Herlander Azevedo

**Affiliations:** 1 Biosystems & Integrative Sciences Institute (BioISI), Plant Functional Biology Center (CBFP), University of Minho, Campus de Gualtar, Braga, Portugal; 2 Area de Genética, Instituto de Hortofruticultura Subtropical y Mediterránea “La Mayora”, Universidad de Málaga-Consejo Superior de Investigaciones Científicas (IHSM-UMA-CSIC), Campus Teatinos, Málaga, Spain; 3 CIBIO, InBIO—Research Network in Biodiversity and Evolutionary Biology, Universidade do Porto, Campus Agrário de Vairão, Vairão, Portugal; 4 PRPlants Lab, GPlantS Unit, Instituto de Tecnologia Química e Biológica—Universidade Nova de Lisboa, Estação Agronómica Nacional, Oeiras, Portugal; 5 Instituto de Investigação e Inovação em Saúde (i3S), Universidade do Porto, Porto, Portugal; 6 Instituto de Biologia Molecular e Celular (IBMC), Universidade do Porto, Porto, Portugal; 7 Instituto de Ciências Biomédicas de Abel Salazar (ICBAS), Universidade do Porto, Porto, Portugal; 8 Departamento de Biologia, Faculdade de Ciências, Universidade do Porto, Porto, Portugal

**Keywords:** Plant development, PTM, SIZ1, sumoylation, SUMO proteases, transcriptome, ULP2

## Abstract

Post-translational modifiers such as the small ubiquitin-like modifier (SUMO) peptide act as fast and reversible protein regulators. Functional characterization of the sumoylation machinery has determined the key regulatory role that SUMO plays in plant development. Unlike components of the SUMO conjugation pathway, SUMO proteases (ULPs) are encoded by a relatively large gene family and are potential sources of specificity within the pathway. This study reports a thorough comparative genomics and phylogenetic characterization of plant ULPs, revealing the presence of one ULP1-like and three ULP2-like SUMO protease subgroups within plant genomes. As representatives of an under-studied subgroup, Arabidopsis *SPF1* and *SPF2* were subjected to functional characterization. Loss-of-function mutants implicated both proteins with vegetative growth, flowering time, and seed size and yield. Mutants constitutively accumulated SUMO conjugates, and yeast complementation assays associated these proteins with the function of *ScUlp2* but not *ScUlp1*. Fluorescence imaging placed both proteins in the plant cell nucleoplasm. Transcriptomics analysis indicated strong regulatory involvement in secondary metabolism, cell wall remodelling, and nitrate assimilation. Furthermore, developmental defects of the *spf1-1 spf2-2* (*spf1/2*) double-mutant opposed those of the major E3 ligase *siz1* mutant and, most significantly, developmental and transcriptomic characterization of the *siz1 spf1/2* triple-mutant placed SIZ1 as epistatic to SPF1 and SPF2.

## Teaser

SPF1 and SPF2 are nuclear-located SUMO proteases that contribute to balancing the levels of SUMO conjugates downstream of SIZ1 and are involved in the regulation of plant development.

## Introduction

Post-translational modifications (PTMs) are able to rapidly and reversibly reprogram protein activity, and are involved in development and responses to environmental challenges. Among the many types of PTMs, one of the most well documented mechanisms is the attachment of small peptides structurally similar to ubiquitin (ubiquitin-like peptides, UBLs) (Miura and Hasegawa, 2010; Vierstra, 2012). Small ubiquitin-like modifier (SUMO) is a UBL family member that is mainly involved in nuclear-associated functions such as the regulation of transcription, chromatin-remodelling, mRNA biogenesis, nuclear–cytoplasm trafficking, and DNA repair (Gareau and Lima, 2010; Mazur and van den Burg, 2012; Cubeñas-Potts and Matunis, 2013). Briefly, sumoylation is achieved by an enzymatic cascade that involves maturation of the pre-SUMO peptide by specific SUMO endopeptidases, followed by three enzymatic steps (SUMO E1 activation, E2 conjugation, and E3 ligation) that drive the transfer of the maturated SUMO to a specific lysine residue, normally within the consensus ψKXE (ψ, large hydrophobic residue; K, lysine; X, any amino acid; E, glutamic acid) (Gareau and Lima, 2010; Cappadocia and Lima, 2018). The attachment can be reversed by specific SUMO isopeptidases, which counteract sumoylation and also contribute to the recycling of the SUMO peptide (Hickey *et al.*, 2012).

SUMO conjugation can exert different effects on a target protein: (1) changing conformation, (2) aiding in protein–protein interactions (PPIs) via SUMO-interacting motifs (SIMs), and (3) blocking of PPIs, for example by competing with other PTMs (Wilkinson and Henley, 2010). Target proteins can be the subject of mono-sumoylation, poly-sumoylation (SUMO chain formation), or multi-sumoylation (multiple sumoylated sites) (Hickey *et al.*, 2012; Hendriks and Vertegaal, 2016). Specificity of sumoylation may be determined by the large number of SUMO proteases, rather than being determined by the conjugation machinery, which is usually encoded by a limited number of genes. SUMO-specific proteases that belong to the C48 family of Cys proteases have been annotated as Ubiquitin-Like protein-specific Proteases or Sentrin/SUMO-specific Proteases (ULPs/SENPs) (van der Hoorn, 2008). These have been described as modulators of sumoylation through their action on SUMO moieties, namely by (1) processing the pre-SUMO (maturation), (2) removing SUMO from the modified target proteins (SUMO deconjugation), or (3) editing SUMO chains. ULP/SENP cysteine proteases are a heterogeneous family, which contribute to the specificity and complexity of the SUMO machinery (Hickey *et al.*, 2012).

In plants, sumoylation seems to be essential for embryonic development, organ growth, flowering transition, and hormone regulation (Elrouby, 2015). In addition, SUMO plays a role in stress-associated responses to stimuli such as extreme temperatures, drought, salinity, and nutrient assimilation (Castro *et al.*, 2012, 2015). During such stresses, the profile of SUMO-modified proteins changes dramatically, with greatly increased SUMO-conjugate levels and a decreased pool of free SUMO (Miller *et al.*, 2013). After the imposition of stress, SUMO conjugates slowly diminish by the action of ULPs. ULPs are fundamental players in the fine-tuning of the SUMO conjugation/deconjugation levels and, consequently, are essential to balance plant growth and stress responses (Conti *et al.*, 2014; Yates *et al.*, 2016). On the other hand, knowledge regarding the importance and functions of SUMO proteases in plant physiology is very limited and many ULPs are yet to be extensively characterized. ULPs fall into two large groups (ULP1s and ULP2s), by homology to yeast ScULP1 and ScULP2. The Arabidopsis genome includes eight predicted ULPs, six of which have been shown to function as SUMO proteases *in vitro* (Chosed *et al.*, 2006; Colby *et al.*, 2006; Conti *et al.*, 2008; Novatchkova *et al.*, 2012; Kong *et al.*, 2017; Liu *et al.*, 2017*a*). Each of these ULPs is likely to contribute individually to specific functions within the plant, judging from the functional characterizations available to date. For instance, ESD4 loss-of-function results in a pleiotropic phenotype (severe dwarfism), while the closely related ELS1 does not have such a severe phenotype (Murtas *et al.*, 2003; Hermkes *et al.*, 2011). OTS1 and OTS2 act redundantly in flowering transition, plant growth, and photomorphogenesis, as well as in pathogen defence, and salt and osmotic stress responses (Conti *et al.*, 2008, 2014; Sadanandom *et al.*, 2015; Bailey *et al.*, 2016; Castro *et al.*, 2016). The function of SPF1 (also designated ASP1) and SPF2 has been recently associated with the control of flowering time, and gamete and embryo development (Kong *et al.*, 2017; Liu *et al.*, 2017*a*).

In the present study, we performed a structural and phylogenetic characterization of plant ULPs, which pointed to SPF1 and SPF2 forming a key subgroup within ULP2-like SUMO proteases. Complementation assays indicated that Arabidopsis *SPF2* is functionally homologous to the yeast *ScULP2* gene and that *SPF1* exerted a dominant negative effect, while *SPF* mutant plants constitutively accumulated more SUMO conjugates. Accordingly, we demonstrate that the SPF1 and SPF2 catalytic domains reacted with SUMO activity-based probes. Arabidopsis T-DNA insertion mutants showed diverse developmental defects, and microarray analysis provided evidence for a specific transcriptional signature that suggests the involvement of SPF1/2 in secondary metabolism, cell wall remodelling, and nitrate assimilation. The *spf1-1 spf2-2* (*spf1/2*) double-mutant also displayed an antagonistic morphological phenotype with respect to the well-characterized SUMO E3 ligase mutant *siz1*. Most significantly, the *spf1/2 siz1* triple-mutant was phenotypically *siz1*-like, which places SPF1/2 as epistatic and downstream of SIZ1.

## Materials and methods

### Plant material and growth conditions

T-DNA insertion mutants were used to evaluate loss-of-function in *Arabidopsis thaliana* SUMO proteases *SPF1/ASP1/ULP2b* (At1g09730) and *SPF2/ULP2a* (At4g33620). Mutants were obtained through the NASC European Arabidopsis Stock Centre (http://arabidopsis.info) or the Arabidopsis Biological Resource Center (https://abrc.osu.edu). All mutants were SALK lines in the background ecotype Columbia-0 (Col): SALK_040576 (*spf1-1*), SALK_022079 (*asp1-2*; also designated as *ulp2like2-2* by Liu *et al.*, 2017*b*), SALK_090744 (*spf2-2*), SALK_140824 (*spf2-3*), SALK_023493C (*spf2-1*), and the previously characterized line SALK_065397 (*siz1-2*). Single T-DNA mutant lines were inter-crossed to obtain the corresponding combination of double-mutants. The *spf1/2 siz1* triple-mutant was obtained by crossing the double-mutant *spf1-1 spf2-2* (i.e. *spf1/2*) with *siz1-2*. The genotypes were confirmed by diagnostic PCR, following the instructions for SIGnAL T-DNA Primer Design (http://signal.salk.edu/tdnaprimers.2.html) and using the primers listed in Supplementary Table S1 at *JXB* online. Synchronized seeds were stratified for 3 d at 4 °C in the dark. Surface-sterilization was performed in a horizontal laminar-flow chamber by sequential immersion in 70% (v/v) ethanol for 5 min and 20% (v/v) commercial bleach for 10 min before washing five times with sterile ultra-pure water. Seeds were resuspended in sterile 0.25% (w/v) agarose, sown onto 1.2% (w/v) agar-solidified MS medium (Murashige and Skoog, 1962) containing 1.5% (w/v) sucrose, 0.5 g l^−1^ MES, pH 5.7, and grown vertically in culture rooms with a 16/8 h light/dark cycle under cool white light (80 µE m^−2^ s^−1^) at 23 °C. For standard growth, 7-d-old plate-grown seedlings were transferred to a soil/vermiculite (4:1) mixture and maintained under identical growth conditions, with regular watering. Mutant lines were morphologically characterized according to the developmental map for Arabidopsis described by Boyes *et al.* (2001). Morphological parameters were measured using the ImageJ software (https://imagej.nih.gov/ij/).

### Pigment extraction and quantification

For estimation of the chlorophyll and carotenoid contents, plant leaves were incubated in 80% (v/v) acetone for 1 h in the dark. The plant material was spun down and absorbances at 470, 645, and 663 nm were measured in a microplate spectrophotometer (SpectraMax 340PC; Molecular Devices). Total chlorophyll was calculated as 20.2A_645_+8.02A_663_ and total carotenoids were calculated as [1000A_470_−1.82(12.7A_663_−2.69A_645_)−85.02(22.90A_645_−4.68A_663_)]/198 (Arnon, 1949; Lichtenthaler and Buschmann, 2001).

Anthocyanin extraction and quantification was adapted from Ticconi *et al.* (2001). Plant leaves were weighed (fresh weight, FW) and incubated at 100 °C for 5 min in extraction buffer composed of 1-propanol (37%, v/v), HCl, and H_2_O, in a 18:1:81 ratio. Samples were subsequently incubated overnight at room temperature in the dark. The plant material was spun down and absorbance of the supernatant was measured at 535 nm and 650 nm in a similar microplate spectrophotometer. Total anthocyanins were calculated as A_535_−A_650_ g^−1^ FW.

### RNA extraction, cDNA synthesis, and RT-qPCR

For reverse-transcription quantitative real-time PCR (RT-qPCR) analysis, RNA from plant tissue was extracted using an RNeasy Plant Mini Kit (Qiagen). RNA quantity and quality were assessed using both a Nanodrop ND-1000 spectrophotometer and standard agarose-gel electrophoretic analysis, and RNA samples were treated with Recombinant DNase I (Takara Biotechnology). Synthesis of cDNA was performed using SuperScript II Reverse Transcriptase Kit (Invitrogen). SsoFast EvaGreen Supermix (Bio-Rad) was used in the RT-qPCR reaction mixture according to the manufacturer’s indications. The reaction was performed in a MyiQ Single-Color Real-Time PCR Detection system (Bio-Rad). Primers for semi-quantitative RT-PCR and RT-qPCR (Supplementary Table S2) were designed using NCBI Primer-BLAST (www.ncbi.nlm.nih.gov/tools/primer-blast/) (Ye *et al.*, 2012) to ensure specific amplification within the Arabidopsis genome, and obeyed the following guidelines: 100–250 bp PCR amplification product size; 50–60% GC content; ~60 °C T_m_. Primers were designed to span an exon junction when possible. *ACT2* (At3g18780) was used as a reference gene (Lozano-Durán *et al.*, 2011).

### Microarray analysis

Genome-wide transcription studies were performed using an ATH1 microarray chip (Affymetrix) with three independent replicates per genotype, with each replicate representing RNA from a pool of four different MS plates containing 10-d-old seedlings. Plants were grown in a plant growth chamber with 16/8 h light/dark cycle under cool white light (80 µE m^−2^ s^−1^) at 21 °C. RNA was extracted as described above, followed by a column cleaning step using an RNeasy Plant Mini Kit (Qiagen). Microarray execution and differential expression analysis were conducted at the Unité de Recherche en Génomique Végétale (Université d’Evry Val d’Essonne, France). The method to determine differentially expressed genes (DEGs) was based on variance modelization by common variance of all genes (Gagnot *et al.*, 2008).

### Plant protein extraction and western blotting

Plant tissue was ground in a microtube in liquid nitrogen with the help of polypropylene pestles. Protein extracts were obtained by adding extraction buffer [50 mM Tris; 150 mM NaCl; 0.2% (v/v) Triton X-100] supplemented with Complete Protease Inhibitor Cocktail (Roche) according to the manufacturer’s instructions. Following incubation with agitation for 1 h at 4 °C, the microtubes were centrifuged twice for 30 min at 16000 *g*. The supernatants were recovered and stored at −80 °C. Protein was quantified spectrophotometrically using Bradford reagent (Sigma; Bradford, 1976). Equal amounts of protein were resolved by standard SDS-PAGE in a 10% (w/v) acrylamide resolving gel, using a Mini-PROTEAN Cell apparatus (Bio-Rad). For western blotting, proteins were transferred to a PVDF membrane using a Mini Trans-Blot Cell (Bio-Rad). The membrane was blocked for 1 h at 23 °C in blocking solution [5% (w/v) dry milk powder in PBST]. The primary antibody anti-AtSUMO1 (Abcam) was added in a 1:1000 dilution and incubated for 3 h. The membrane was washed three times with 10 ml of PBST for 10 min, and then incubated with the secondary anti-rabbit antibody (Santa Cruz) at 1:2000 in blocking solution for 1 h. The membrane was washed as described above and developed using a chemiluminescence reaction with an Immune-Star WesternC Kit (Bio-Rad) and a ChemiDoc XRS system (Bio-Rad) for image acquisition. PVDF membranes were incubated for 15 min with Ponceau S solution [0.1% (w/v) Ponceau S; 5% (v/v) acetic acid] to stain for total proteins.

### Plasmid construction

Arabidopsis *SPF1* and *SPF2* coding-sequence (CDS) PCR products were purified and cloned using the pGEM-T Easy system (Promega). Final constructs for *pGEM-SPF1* and *pGEM-SPF2* were confirmed by sequencing. The *SPF1* sequence was shorter than the one annotated in TAIR (www.arabidopsis.org), implying the existence of two additional introns. This shorter SPF1 isoform sequence displayed a complete match with the protein sequence NP_001184951.1 in the NCBI database (http://www.ncbi.nlm.nih.gov/). The *SPF1* and *SPF2* full fragments were excised by restriction using *Not*I and *Asc*I and were then subcloned into the Gateway Entry vector *pENTR*. The LR reaction for recombination between the *att*L (entry clone) and *att*R (destination vector) recombination sites was carried out in the *pMDC43* vector (Curtis and Grossniklaus, 2003). Recombinations between the *pENTR* constructs and the *pMDC43* destination vector were performed using LR Clonase II (Invitrogen), following the manufacturer’s instructions.

To generate *pCM190-SPF2*, primers with the restriction sites *Pme*I-*Not*I (Supplementary Table S3) were used to amplify the *SPF2* CDS from *pGEM-SPF2* and, after digestion with *Pme*I-*Not*I, the product was subcloned into *pCM190*. *pGEM-SPF1* was digested with *Not*I and the resulting fragment was cloned into *pCM190* to yield *pCM190-SPF1*.

The cDNAs encoding the catalytic domains of *SPF1* and *SPF2* (hereafter referred to as *cSPF1* and *cSPF2*) were amplified from *pGEM-SPF1* and *pGEM-SPF2* using primers listed in Supplementary Table S3. Amplification products were cloned into *pNZY28-A* using the NZY-A PCR cloning kit (NZYtech). *cSPF1* and *cSPF2* were then respectively excised using the restriction enzyme combinations *Eco*RI + *Not*I and *Bam*HI + *Not*I (NEB) to clone into the expression vector *pGEX-5X-1* (GE Healthcare).

### Yeast complementation assay

The yeast (*Saccharomyces cerevisiae*) mutant strain *ulp1-ts* (temperature-sensitive) has been described previously (Li and Hochstrasser, 1999), and the *ulp2∆* mutant and an isogenic *ULP2*^*+*^ strain were obtained by sporulation of the EUROSCARF diploid strain (*Y21424*, *Mat α/a*; *his3D1/his3D1*; *leu2D0/leu2D0*; *lys2D0/LYS2*; *MET15/met15D0*; *ura3D0/ura3D0*; *YIL031w::kanMX4/YIL031w*). Both mutant strains were used for the complementation assays with *SPF1* and *SPF2* from Arabidopsis. Yeast strains *ulp1-ts* and *ulp2∆* were transformed (Gietz *et al.*, 1995) with the constructs *pCM190-SPF1*, *pCM190-SPF2*, or the empty vector (*pCM190*), and plated at 25 °C on minimal medium (yeast nitrogen base, YNB) supplemented with the appropriate amino acids according to each strain genotype, and doxycycline (10 g ml^−1^; Sigma). The constructs expressed *SPF1* or *SPF2* from a tetracycline-regulatable promoter, so the tetracycline analogue doxycycline was added to the plates to inhibit *SPF1* or *SPF2* expression (Garí *et al.*, 1997). A 10-fold serial dilution of three independent colonies for each transformation was made, and 5 μl of each dilution was spotted onto minimal medium (YNB) supplemented with the appropriate amino acids with or without doxycycline (10 g ml^−1^). The plates were incubated at 25 °C or 37 °C for 5 d.

### Covalent labelling with HA-tagged HsSUMO-VME probes

Vinyl methyl esters (VMEs) are probes that react irreversibly with the ULP catalytic cysteine and establish a covalent bound that can be detected by SDS-PAGE followed by western blotting using an anti-HA antibody (Borodovsky *et al.*, 2002). Recombinant glutathione S-transferase (GST)-SPF expression constructs were transformed into the *E. coli* strain BL21(DE3) pLysS, and expression was induced at an A_600_ of 0.6 with 0.1 mM IPTG at 16 °C overnight. Cells were harvested by centrifugation for 20 min at 4000 *g*. Bacterial pellets were resuspended in buffer A (50 mM Tris-HCl, pH 8.0; 150 mM NaCl; 1 mM BME), disrupted by sonication, and cleared by centrifugation at 34000 *g* for 40 min. Recombinant GST-SPF protein was purified by batch affinity chromatography using Glutathione Agarose beads (ThermoFisher Scientific). The beads were washed with buffer A, and proteins were eluted on gravity columns with buffer B (50 mM Tris-HCl, pH 8.0; 10 mM reduced L-glutathione). Eluted proteins were stored at –80 °C.

The SPF activity assay was performed in a reaction buffer containing 50 mM Tris-HCl, pH 8.0, 150 mM NaCl, 10% (w/v) glycerol, 2 mM EDTA-NaOH, 0.15 µg µl^−1^ BSA, and 2 mM DTT (Pinto *et al.*, 2012). Human SENP1 catalytic domain (cSENP1) and UCHL3 were obtained as described by Pinto *et al.* (2012) and Grou *et al.* (2015), respectively. Human SUMO (HsSUMO) probes (Borodovsky *et al.*, 2002) were obtained as described by Pinto *et al.* (2012). The reaction was carried out at 25 °C for 2 h, using 100 ng of human influenza hemagglutinin (HA)-HsSUMO-VME and 200 ng of either protease in a final volume of 20 µl. The reaction sample was mixed with Laemmli sample buffer and incubated at 65 °C for 10 min followed by 5 min at 95 °C. Proteins were separated electrophoretically in a 16% SDS-PAGE gel, transferred to a nitrocellulose membrane, and probed with monoclonal anti-HA antibody (16B12, Covance) and phosphatase alkaline-conjugated secondary antibody anti-mouse IgGs (A2429, Sigma-Aldrich).

### Transient expression in tobacco

*Agrobacterium tumefaciens* EHA105 containing constructs-of-interest was co-infiltrated with a suppressor of gene silencing, the p19 protein of tomato bushy stunt virus (TBSV), to prevent the onset of post-translational gene silencing (Silhavy *et al.*, 2002). The pellet was resuspended in 1 ml agroinfiltration buffer [10 mM MgCl_2_; 10 mM MES, pH 5.6; 19.6 mg ml^−1^ acetosyringone] and grown in non-supplemented medium until a final A_600_ of 1 was obtained for the empty or transformed strain, and A_600_ of 2 for the *p19* silencing vector. The resuspended pellets of both the transformed strain and *p19* were incubated for 2–5 h and subsequently infiltrated in a 1:1 ratio with a 5-ml syringe in the abaxial side of 3-week-old *Nicotiana benthamiana* leaves. Expression of each transgene was monitored 4 d after transformation with an Olympus FluoView FV1000 confocal laser microscope, using excitation wavelengths of 488 nm (green fluorescent protein, GFP) and 635 nm (chloroplast autofluorescence). Bright-field images were detected using transmitted light. Detection specifications were maintained between different biological samples.

### Phylogenetic and bioinformatics analysis

The automated gene family annotation resource Plaza (Van Bel *et al.*, 2012) was used to retrieve amino acid sequences of ULP gene family members across 30 phylogenetically representative species, based on queries using the search terms At4g15880, At1g09730, At1g60220, and At3g48480. Phylogenetic analysis was performed using maximum likelihood (RaxML) with 1000 bootstrap iterations, as previously described (Castro *et al.*, 2017). The final output of the tree was produced using the SeaView v4.4.0 software (Gouy *et al.*, 2010). Protein sequence alignment of the catalytic domain of Arabidopsis SPFs with homologous proteins from eukaryotic organisms was performed using PRALINE (Simossis and Heringa, 2005). Gene ontology (GO) term functional categorization was performed in VirtualPlant 1.2 (http://virtualplant.bio.nyu.edu/cgi-bin/vpweb/) using the BioMaps function with a 0.05 *P*-value cut-off (Katari *et al.*, 2010). Redundancy exclusion and scatterplot analysis were performed using REVIGO (http://revigo.irb.hr/), with a 0.7 C-value. The scatterplot presents the cluster representatives in a two-dimensional space (*x*- and *y*-axis) derived by applying multidimensional scaling to a matrix of the semantic similarities of the GO terms (Supek *et al.*, 2011). MapMan was used to plot *spf1/2* deregulated genes in the Metabolism overview pathway map (http://mapman.gabipd.org/web/guest/home) (Thimm *et al.*, 2004).

## Results

### Plant ULP2 proteases are phylogenetically and topologically diverse

**Fig. 1.**
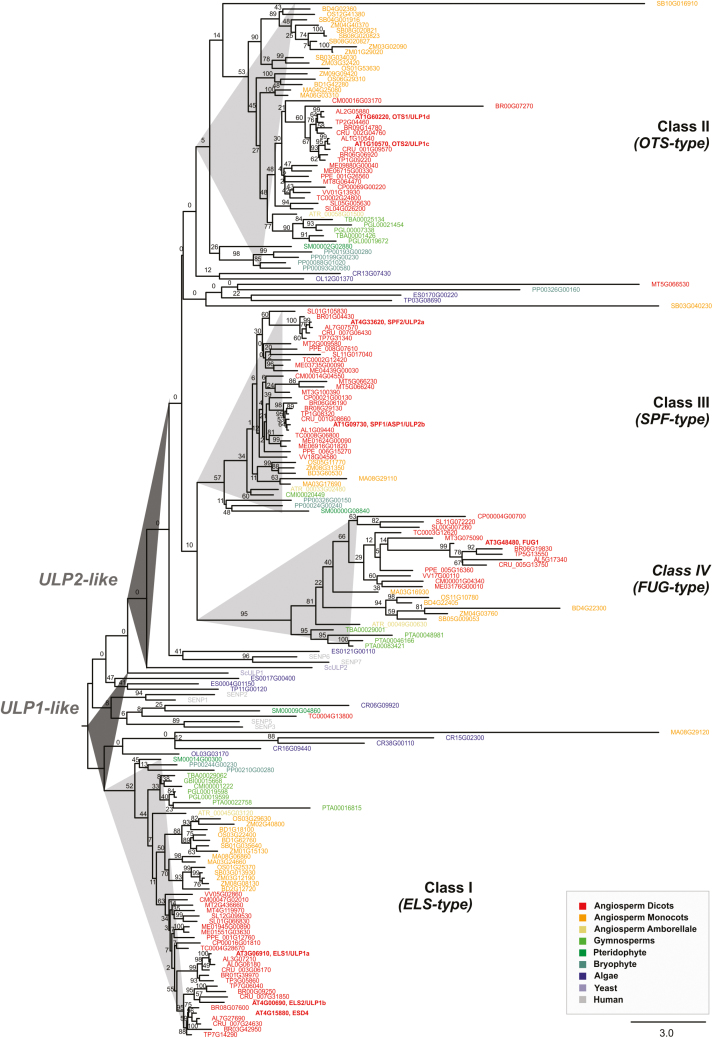
Phylogenetic analysis of the plant ubiquitin-like protease (ULP) family. The phylogenetic reconstruction includes ULPs present in representative plant genomes, as well as human SENPs and yeast (*Saccharomyces cerevisiae*) ULPs. Phylogenetic analysis was performed using maximum-likelihood with bootstrap analysis (1000 trees; numbers on each branch represent the percentages of bootstrap).

Previous predictions for Arabidopsis ULP SUMO protease family members have been scarce in scope and, above all, inconsistent as to the relationships between the main existing phylogenetic subgroups. For instance, they have missed inclusion of the At3g48480 protein, or placed OTS1 and OTS2 (also termed ULP1d and ULP1c) in either ULP1- or ULP2-related clades (Miura *et al.*, 2007*a*; Lois, 2010; Novatchkova *et al.*, 2012). To resolve this issue, we performed a significantly more comprehensive ULP phylogeny. A plant ULP ortholog search in 30 representative genomes was carried out using Plaza (Proost *et al.*, 2015), and was based on homology searches with the seven consistently annotated Arabidopsis ULPs and the putative family member At3g48480. The phylogenetic reconstruction displayed two major branches that resolved ULP1s (yeast *ScULP1* and human *SENP1-3*, *-5*) and ULP2s (yeast *ScULP2* and human *SENP6-7*) (Fig. 1). Both branches contained algae and plant ULPs from all major taxa, demonstrating the polyphyletic origin of plant ULPs. Our analysis uncovered a series of interesting findings. ULP1s encompassed Arabidopsis ESD4, ELS1 (also termed ULP1a) and ULP1b, whereas Arabidopsis OTS1 and OTS2 are most likely ULP2s and not ULP1s. Plant ULPs could be further categorized into four phylogenetic subgroups or classes (Fig. 1), which we have named based on the classification proposed by Novatchkova *et al.* (2012). Class II (*OTS-type*; OTS1/2) and Class III (*SPF-type*; SPF1/2) contained paralogs from all major taxa all the way to briophytes, suggesting a very ancestral duplication and subsequent subfunctionalization that remained conserved across plant evolution. Arabidopsis ULP At3g48480, which was often absent from ULP annotation (possibly due to its smaller protein size), showed up as an independent subclade/class across at least the flowering plant taxa, and was named Fourth ULP Gene Class 1 (FUG1).

**Fig. 2.**
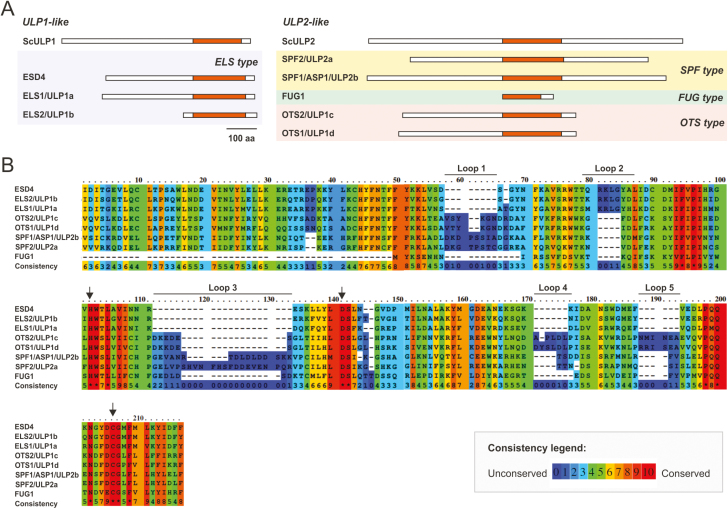
Topological analysis of the plant ULP proteins. (A) Schematic representation of Arabidopsis and yeast ULP protein topology with the catalytic domain highlighted in orange. The scale bar indicates 100 amino acids. (B) Protein sequence alignment of the catalytic domain in Arabidopsis ULPs. The arrows indicate the three conserved catalytic residues. Consistency between sequences indicates the levels of conservation of each residue. Five main extensions can be discriminated within the catalytic domain (loops 1–5).

The present study specifically addressed Arabidopsis *SPF1* (also termed *ULP2b* or *ASP1*; At1g09730) and *SPF2* (also termed *ULP2a*; At4g33620). Their proteins displayed 30.5% identity, as well as a highly conserved region that matched the catalytic domain and possessed 46% identity (Fig. 2A, B, Supplementary Fig. S1). For both proteins, topological analysis revealed the catalytic domain to be located within the centre of the protein, while ULP1-like proteins were located in the C-terminal end (Fig. 2A). Analysis also demonstrated that At3g48480 was restricted to the catalytic domain and lacked both the N- and C-terminal ends of ULP2s (Fig. 2A). Remarkably, the catalytic triad (His-Asp-Cys), essential for protease activity, was conserved among all Arabidopsis ULP members (Fig. 2B). Within the catalytic domain, it was possible to discriminate five main extensions (loops 1 to 5; Fig. 2B). Loops 1, 3, 4, and 5 were common to SPF1/2 and OTS1/2, and absent in ESD4, ELS1, and ULP1b, while loop 2 was specific to ULP1b. Loop 1 and in particular loop 2 were larger in SPF1/2, whereas loops 3 and 4 were larger in OTS1/2 (Fig. 2B).

### SPF1 and SPF2 differentially complement yeast *ulp1* and *ulp2* mutants and react with activity-based SUMO probes

**Fig. 3.**
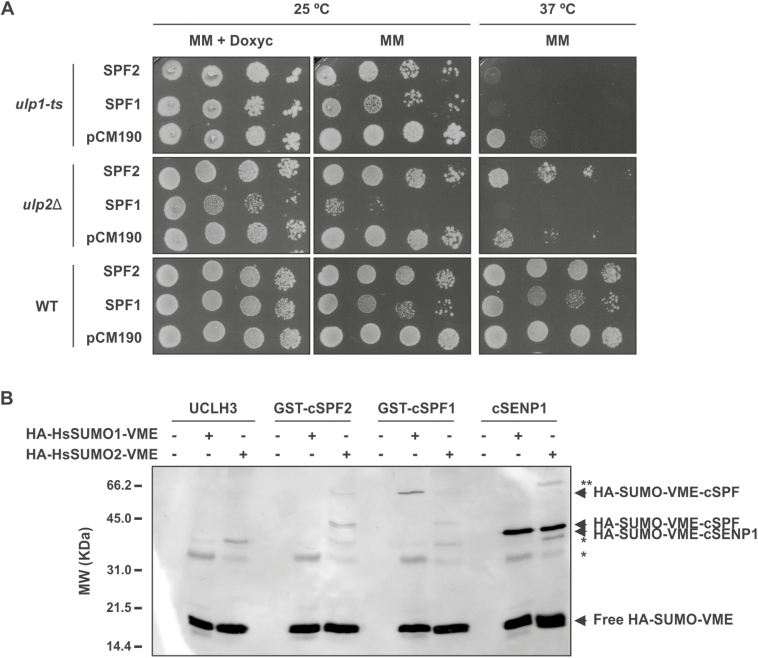
SUMO protease activity analysis of SPF1 and SPF2 by yeast complementation assays and reactivity of SPF1/2 catalytic domains towards human SUMO (HsSUMO) vinyl methyl ester (VME) probes. (A) Transformants harbouring the vector *pCM190* or the constructs to express *SPF1* (*pCM190-SPF1*) and *SPF2* (*pCM190-SPF2*) were plated on selective minimal medium (MM) with doxycycline (10 g l^−1^) and incubated at 25 °C for 4 d. Ten-fold serial dilutions were made for three independent colonies (a representative colony is shown for each transformation) and 5 μl of each dilution was spotted onto MM or selective MM with doxycycline. Plates were incubated at 25 °C or 37 °C as indicated, and photographs were taken after a 5-d incubation. (B) *In vitro* SPF1 and SPF2 catalytic domain (cSPF1 and cSPF2) activity was tested against HA-HsSUMO1-VME and HA-HsSUMO2-VME. Human deubiquitinase UCHL3 and SUMO protease SENP1 catalytic domain (cSENP1) were used as negative and positive control enzymes, respectively. The arrows indicate free HA-HsSUMO-VME probes and their conjugated forms with ULPs, as labelled. * Indicates unspecific bands; ** indicates a possible adduct between an SDS resistant dimer of cSENP1 and SUMO2-VME. Molecular weight markers (MW) are displayed.

SUMO proteases may display different activities, breaking endopeptidic bonds that are important for SUMO maturation or having isopeptidic activity for SUMO removal or SUMO chain editing (Hickey *et al.*, 2012). Phylogenetic analysis placed SPF1 and SPF2 closer to ULP2s from non-plant models (yeast Ulp2 and human SENP6/7; Fig. 1). To validate this hypothesis, yeast complementation of the *ulp1* and *ulp2* mutants was performed, expressing the Arabidopsis genes from a multicopy plasmid (*pCM190*; Garí *et al.*, 1997). Yeast *ULP1* is an essential gene, so the complementation assay required the use of a previously described temperature-sensitive mutant (*ulp1-ts*) (Li and Hochstrasser, 1999). The deletion of the yeast *ULP2* gene is not lethal, but *ulp2Δ* mutants show sensitivity to a variety of stresses, including elevated temperature (Li and Hochstrasser, 2000; Schwienhorst *et al.*, 2000). Hence, the temperature-sensitive phenotype of the *ULP2* deletion allele *ulp2Δ* (Y21424, EUROSCARF) was used for the complementation assay with Arabidopsis SPF1 and SPF2. Both yeast mutants were transformed with the vector (*pCM190*) or the plasmid expressing either SPF1 or SPF2, from a tetracycline-regulatable promoter, so that expression was inhibited in the presence of doxycycline (a tetracycline analogue) (Garí *et al.*, 1997). The temperature-sensitive *ulp1-ts* mutant was not able to grow at 37 °C when any of the two Arabidopsis genes were expressed (Fig. 3A). However, SPF2 could complement *ulp2Δ* temperature sensitivity, whereas SPF1 could not. Remarkably, the *ulp2∆* mutant was sensitive to SPF1 expression and yeast growth was clearly diminished at both temperatures (Fig. 3A). The toxic effect of SPF1 was doxycycline-dependent and more severe in the *ulp2∆* background than in an isogenic wild-type strain or in the *ulp1-ts* mutant (Fig. 3A), suggesting a dominant-negative mutant effect of the presence of Arabidopsis SPF1 in the absence of the yeast *ULP2* ortholog. Collectively, these results suggested that (1) Arabidopsis SPF1 and SPF2 were not ULP1 proteases, (2) *SPF2* was functionally homologous to the yeast *ULP2* gene, and (3) SPF1 function was related to ULP2 SUMO proteases.

To examine SPF1 and SPF2 SUMO protease activity *in vitro*, we used activity-based irreversible inhibitors in the form of vinyl methyl ester (VME)-derivatized HA-tagged HsSUMO1 and HsSUMO2 (HA-HsSUMO1-VME and HA-HsSUMO2-VME, respectively). As previously stated, VME probes bind irreversibly to the ULP catalytic domain, which can be detected by western blotting (Borodovsky *et al.*, 2002). For the activity assay, we expressed and purified the SPF1 and SPF2 catalytic domains (cSPF1 and cSPF2) coupled with a GST-tag at the N-terminus. cSPF2 reacted positively towards HsSUMO2-VME, while cSPF1 reacted mainly with HsSUMO1-VME (Fig. 3B). In addition, SPF2 revealed other bands with lower molecular weight that were probably the result of sub-products of SPF2 expression reacting with HsSUMO2-VME (Supplementary Fig. S2). The negative control UCHL3, a specific protease for ubiquitin, did not react with any of the probes while cSENP1, a human ULP (Kolli *et al.*, 2010), reacted towards human SUMO, revealing a shift for the expected size of SENP1-HsSUMO-VME (Fig. 3B). Collectively, results supported the roles of SPF1 and SPF2 as SUMO proteases.

### SUMO conjugate levels are modulated by SPF1 and SPF2 *in planta*

**Fig. 4.**
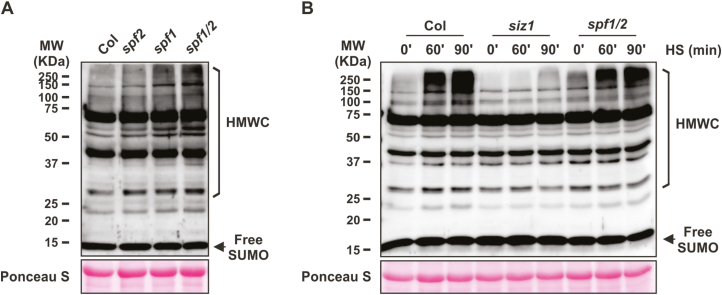
Immunoblot analysis of high molecular weight SUM1 conjugates (HMWC) in Arabidopsis wild-type Col-0 and *SPF* mutants. (A) Analysis of leaf protein extracts from 1-month-old plants grown in soil. (B) Analysis of plate-grown 10-d-old plants subjected to heat-shock (HS, 37 °C) for 0, 60, or 90 min; the *siz1* mutant was used as a negative control of SUMO conjugate induction after heat shock. Protein extracts (50 µg per lane) were analysed by immunoblotting using anti-AtSUMO1 polyclonal antibodies. The larger subunit of Rubisco stained with Ponceau S was used as a loading control. Molecular weight markers (MW, *Kaleidoscope*, Bio-Rad) are displayed.

To verify whether SPF1 and SPF2 had an impact on SUMO-conjugate levels, we examined the sumoylation profiles in Arabidopsis SPF1 and SPF2 T-DNA insertion lines (Supplementary Fig. S3A). Given that SPF1 and SPF2 are phylogenetically and topologically close (Figs 1, 2) and that functional redundancy has been displayed by other gene family members (Castro *et al.*, 2016), we also generated a double-mutant *spf1-1 spf2-2* (hereafter referred to as *spf1/2*). We confirmed abolished gene expression in the mutant backgrounds using semi-quantitative RT-PCR (Supplementary Fig. S3B). Sumoylation patterns were analysed by western blotting of whole-plant protein extracts using specific anti-AtSUMO1 antibodies, thus covering the predominant SUMO1/2 peptides (Saracco *et al.*, 2007; van den Burg *et al.*, 2010). When compared to the Col-0 wild-type, high molecular weight SUMO conjugates constitutively accumulated in the *spf1/2* double-mutant and also to some extent in the single mutants (Fig. 4A). To further characterize the lack of SPF1 and SPF2 in Arabidopsis, we examined the level of SUMO conjugates of the Arabidopsis *spf1/2* double-mutant subjected to heat-shock (HS) stress (Fig. 4B). SUMO-conjugation increased in response to stress, and this increment could be regulated by an altered balance between conjugation and deconjugation activities, in which ULPs play an important role (Pinto *et al.*, 2012). Here, although HS stress induced SUMO-conjugate accumulation, no major changes were observed in *spf1/2* compared to the wild-type, as the conjugate levels in Col-0 in response to HS were close to those in the conjugate-overproducer *spf1/2* background. As expected, SUMO conjugates failed to accumulate in the *siz1* mutant that was used as a negative control.

### SPF1 and SPF2 are localized in the nucleus

**Fig. 5.**
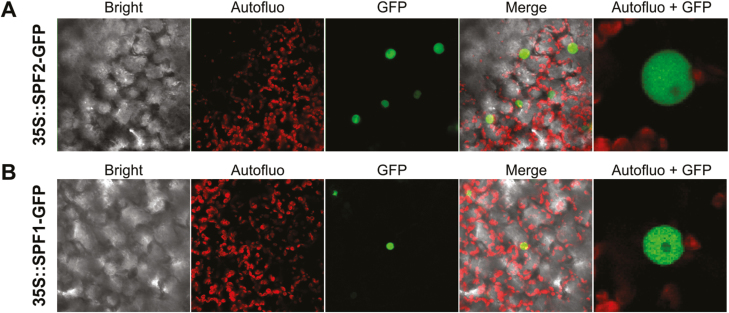
Subcellular localization of Arabidopsis SPF1 and SPF2. SPF2 (A) and SPF1 (B) were N-terminally fused to GFP and transiently expressed in *N. benthamiana* leaves. The confocal microscopy channels depict a 60× magnification of bright field, chloroplast autofluorescence (red), GFP fluorescence (green), or an overlay of these channels (Merge). Autofluo+GFP represents a digital magnification of the cell nucleus.

Differential recognition of SUMO substrates by SUMO proteases has been partially attributed to differences in subcellular localization (Hickey *et al.*, 2012). Since localization of ULP proteins is crucial for their biological function, we investigated the location of SPF1 and SPF2 within the plant cell using transient expression of GFP-fusion proteins in *N. benthamiana*. Expression was visualized by confocal microscopy 3 d after agroinfiltration. Both SPF1 and SPF2 were localized specifically within the nucleus (Fig. 5A, B), showing no signal at the nucleolus, which was suggestive of specific subnuclear localization for both proteins.

### SPF1 and SPF2 mutants are developmentally compromised

**Fig. 6.**
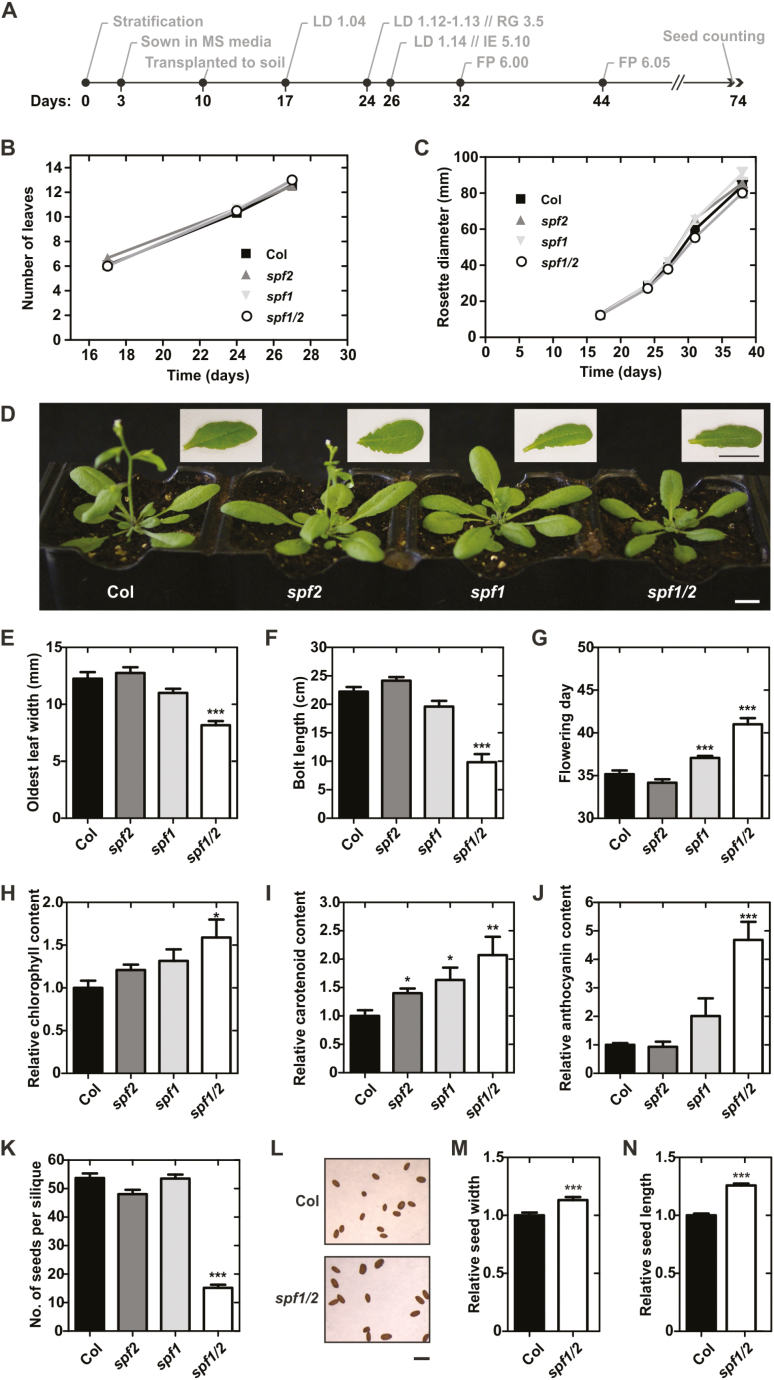
Developmental characterization of Arabidopsis wild-type Col-0, and the *spf1*, *spf2*, and *spf1/2* mutants. (A) Chronological scheme of Col-0 development, with selected stages based on phenotypic analysis of soil-grown plants (Boyes *et al.*, 2001); LD, leaf development; RG, rosette growth; IE, inflorescence emergence; FP, flower production. Number of leaves (B) and rosette size (C) of plants at key developmental stage points. (D) Morphology of 1-month-old plants grown under long days. Insets show a representative leaf of each genotype. Scale bars indicate 1 cm. (E, F) Morphological measurements of 1-month-old plants. (G) Age of plants at flowering. Chlorophyll (H), carotenoid (I) and anthocyanin (J) contents in 1-month-old plants relative to Col-0. (K) Number of seeds per silique. (L) Seed morphology in Col-0 and the *spf1/2* mutant; the scale bar indicates 1 mm. Seed width (M) and length (N) relative to Col-0. Error bars represent standard error of the means (SEM): *n*=12 (B, C, E–G); *n*=6 (H, I); *n*=5 (J); *n*=6 (K); and *n*>36 (M, N). Significant differences with respect to the wild-type were determined using unpaired *t*-tests *P<0.05; **P<0.01; ***P<0.001.

Sumoylation has been shown to modulate many aspects of plant development, as well as key mechanisms in various stress responses. Many of the previous findings regarding the role of SUMO in plants have been based on reverse genetics approaches (Lois, 2010). To investigate SPF1 and SPF2 function, a systematic characterization of morphological/developmental features of null-mutants was carried out (Fig. 6A). In the earlier stages of development there were no severe phenotypic differences between genotypes growing in soil (Fig. 6B, C). However, at later developmental stages, morphological analysis suggested that, in comparison to Col-0, both the *spf1* and *spf1/2* mutants displayed altered leaf morphology and late flowering times (Fig. 6D). Although *spf1/2* rosettes displayed a slightly smaller diameter (not significantly different), the most interesting aspect was that *spf1/2* leaves were significantly smaller in width (Fig. 6E). Overall, *spf1/2* plants showed a clear delay in development that included late flowering and a shorter bolt length at that developmental point (Fig. 6F, G), but taller plants at the end of the life cycle (Supplementary Fig. S4A). Another striking feature of the double-mutant plants was the darker colour of the leaves, and hence we measured pigment contents in leaves of 1-month-old plants (Fig. 6H–J). The results indicated that *spf1/2* accumulated relatively more chlorophyll, carotenoids, and anthocyanins than Col-0. Finally, we observed that *spf1/2* seed production and morphology were also severely affected, resulting in a low number of seeds per silique (Fig. 6K), but seeds were bigger compared to Col-0 (Fig. 6L–N). No differences were observed for silique size between *spf1/2* and Col-0 (Supplementary Fig. S4B). To genetically confirm the present results, second allele mutants were examined and displayed similar phenotypes (Supplementary Fig. S5). Collectively, the results indicated that the *spf1/2* double-mutant aggravated various single mutant phenotypes, indicating at least partial functional redundancy between SPF1 and SPF2.

**Fig. 7.**
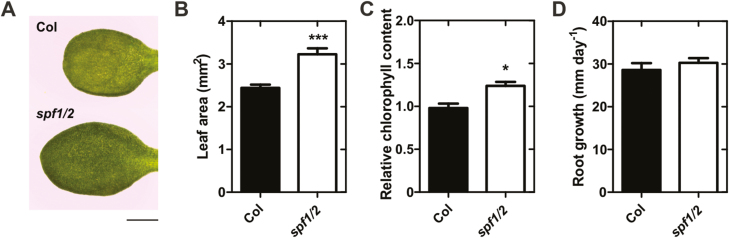
Morphological differences between 10-d-old seedlings of wild-type Col-0 and the *spf1/2* mutant grown on plates. (A) Morphology of one representative leaf of each genotype. Scale bar represents 1 mm. Leaf area (B), relative chlorophyll content (C), and root growth (D). Error bars represent SEM: *n*=9 (B); *n*≥5 (C); and *n*≥12 (D). Significant differences with respect to the wild-type were determined using unpaired *t*-tests: *P<0.05; ***P<0.001.

In addition to the phenotypes displayed in plants growing in soil, we noticed that the leaves of plate-grown, 10-d-old *spf1/2* mutants were bigger and darker than those of the wild-type (Fig. 7A). We therefore characterized *spf1/2* seedlings growing in MS media for 10 d. Compared to the Col-0 wild-type, *spf1/2* seedlings displayed a greater leaf area and higher chlorophyll content (Fig. 7B, C), but no differences were observed for root growth (Fig. 7D). In summary, we observed a series of developmental phenotypes in *spf1/2*, at both earlier and later stages, which revealed that these proteins were important for multiple steps in plant development.

### Microarray analysis implicates SPF1/2 in the control of development and secondary metabolism

**Fig. 8.**
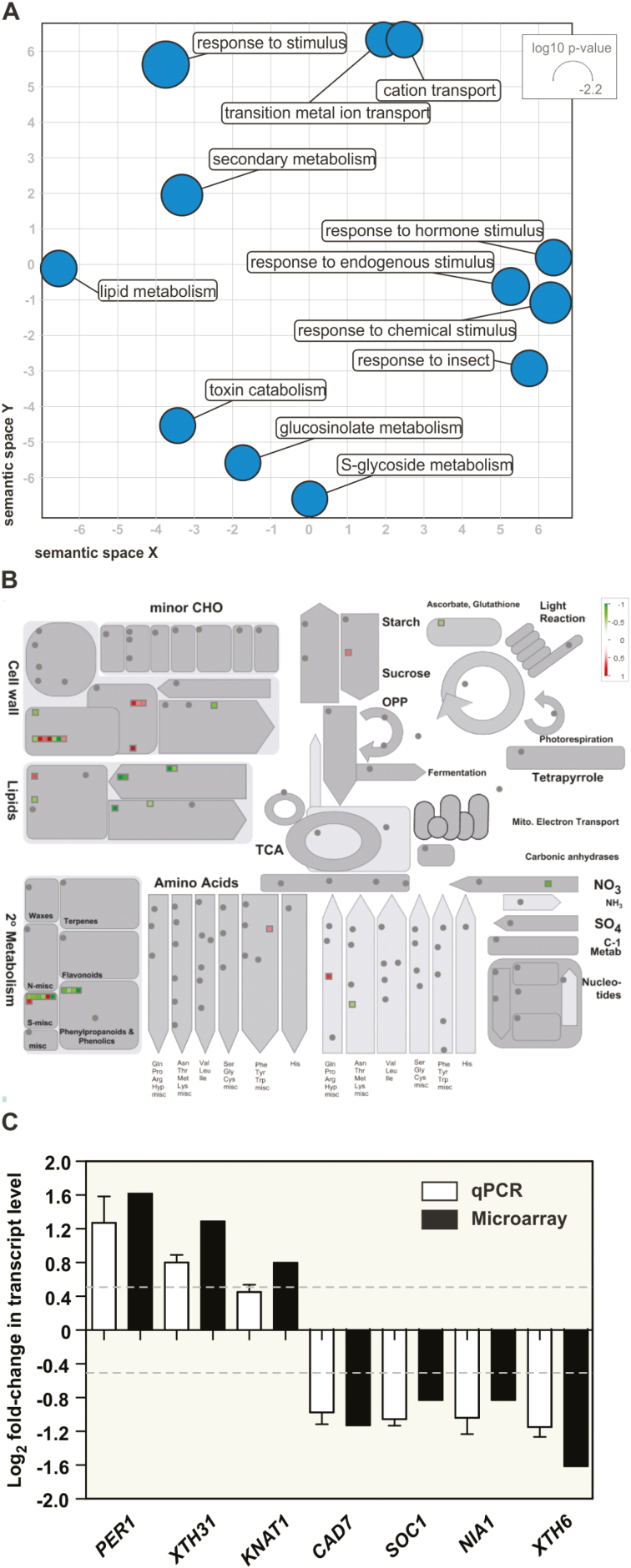
Transcriptomic analysis of 10-d-old plate-grown *spf1/2* seedlings. (A) Scatterplot analysis of enriched gene ontology (GO) terms for *spf1/2* differentially expressed genes. The size of the circles indicates the frequency of the GO term. (B) MapMan analysis of *spf1/2* deregulated genes using the ‘Metabolism overview pathway’ map. The colour gradient indicates down-regulated genes (green) to up-regulated genes (red). (C) RT-qPCR analysis of differentially expressed genes in the *spf1/2* mutant compared to the Col-0 wild-type: *PER1* (At1g48130), *XTH31* (At3g44990), *KNAT1* (At4g08150), *CAD7* (At4g37980), *SOC1* (At2g45660), *NIA1* (At1g77760), and *XTH6* (At5g65730). Error bars represent SEM of three independent biological replicates. The dashed lines represent the threshold for log_2_ fold-change that was used to set differential expression in the microarray experiment.

Sumoylation is strongly involved in nuclear mechanisms, particularly in the control of gene transcription through the regulation of chromatin remodelling complexes, co-repressors, and modulators of transcription factor (TF) activity (Mazur and van den Burg, 2012). In light of this, SPF1 and SPF2 might modulate gene expression by promoting desumoylation and counteracting SUMO-dependent control of transcriptional regulators. To determine whether the transcriptional profile correlated with SPF1/2 function, we performed a microarray analysis of 10-d-old wild-type and *spf1/2* plants. We had already demonstrated the presence of altered plant morphology (Fig. 7) and SUMO conjugate levels (Fig. 4B) at this developmental stage. Microarray analyses indicated that 115 genes were down-regulated and 100 were up-regulated. Gene ontology (GO) and MapMan analyses were used to compare differential expression against biological processes and the overall metabolic pathways of Arabidopsis (Fig. 8A, B). The results revealed that many differentially expressed genes (DEGs) were involved in cell wall and secondary metabolism, including genes pertaining to the biosynthesis of phenylpropanoids (particularly lignin biosynthesis), glucosinolates and lipids (Fig. 8A, B; Table 1). The majority of these genes were found to be down-regulated. In contrast, one GO category particularly enriched in *spf1/2* was the response to hormone stimulus. Although no specific hormone signature could be highlighted, we could observe the up-regulation of genes that are functionally associated with auxin, brassinosteroid, cytokinin, gibberellin, jasmonate, and salicylic acid hormones (Table 1).

Interestingly, some genes previously described as being deregulated in *siz1* mutants were inversely expressed in *spf1/2* DEGs. Examples included nitrate reductase *NIA1* (At1g77760), the AGAMOUS-like transcription factor *SOC1* (At2g45660), and the xyloglucan endotransglucosylase/hydrolase *XTH31* (At3g44990) (Jin *et al.*, 2008; Miura *et al.*, 2010; Park *et al.*, 2011), which are involved in N-assimilation, flowering time, and cell growth, respectively. In *spf1/2*, the observed deregulation in transcript levels for these and other genes was confirmed by RT-qPCR (Fig. 8C), thus validating our microarray data.

Co-expressed genes tend to be controlled by identical transcriptional regulators, and share common *cis*-elements in their promoters. Given that sumoylation often targets regulators of transcription, we identified statistically over-represented *cis*-elements in the promoters of *spf1/2* DEGs that may act as binding sites for SUMO target candidates. In our DEGs, we were able to observe an enrichment in MYC2-like binding sites (Supplementary Table S4) in both up- and down-regulated genes.

### SIZ1 is epistatic to SPF1/2

**Fig. 9.**
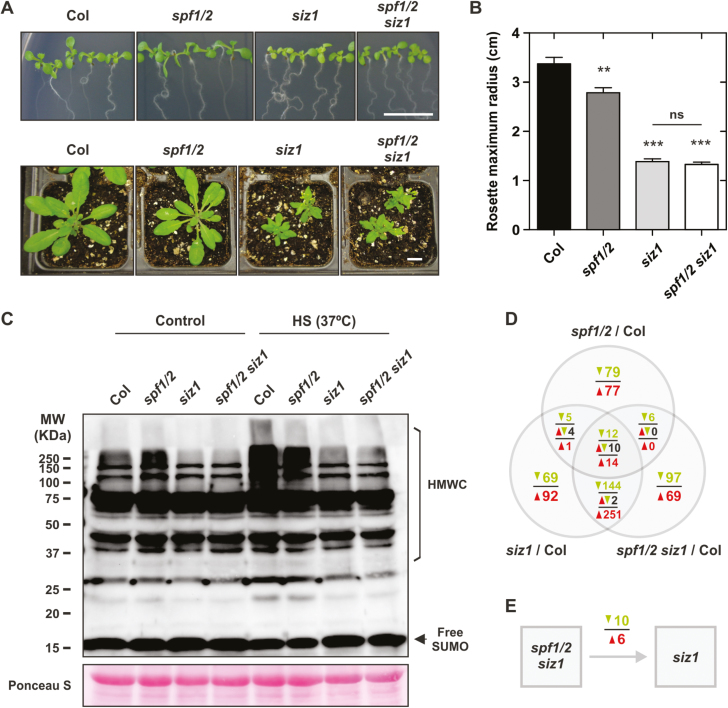
Characterization of the *spf1/2 siz1* triple-mutant. (A) Morphology of 10-d-old plate-grown and 1-month-old soil-grown plants. (B) Rosette maximum radius. Error bars represent SEM, *n*=7. Significant differences for mutants compared to the wild-type Col-0, and for *siz1* compared to *spf1/2 siz1* were determined using unpaired *t*-tests: ns, non-significant; **P<0.01; ***P<0.001). (C) Western blot analysis of high molecular weight SUM1 conjugates (HMWC) in 10-d-old Col-0, *spf1/2*, *siz1*, and *spf1/2 siz1* subjected to heat shock (HS) for 1 h. (D) Venn diagram representing differentially expressed genes in each mutant genotype compared to the wild-type Col-0. (E) Differentially expressed genes in the *spf1/2 siz1* triple-mutant in relation to the single-mutant *siz1*. In (D, E) the colour scheme represents down-regulated genes (green), up-regulated genes (red), and anti-expressed genes (black).

When we compared *spf1/2* to mutants of the Arabidopsis SUMO conjugation pathway, it become clear that *spf1/2* displayed antagonistic phenotypes to those of *siz1*. SIZ1 is the major SUMO E3 ligase and has been the subject of most functional studies in the pathway. In contrast to SPF1/2, loss of SIZ1 function induces diminished accumulation of SUMO conjugates, early flowering, and decreased pigment content (Catala *et al.*, 2007; Jin *et al.*, 2008; P.H. Castro *et al.*, unpublished results), suggesting an epistatic relationship between SIZ1 and SPFs. To further examine this, we generated a *spf1/2 siz1* triple-mutant and determined its phenotype characterization. Morphologically, the triple-mutant resembled *siz1* and was similarly affected in the accumulation of high molecular weight SUMO conjugates, even after HS treatment (Fig. 9A–C), suggesting that SIZ1 was acting upstream of SPF1/2.

Transcript profiling was carried out on the *spf1/2 siz1* triple-mutant, and this was compared to the *siz1* and *spf1/2* transcriptomes (Fig. 9D). We identified DEGs in all three mutant genotypes in comparison to the wild-type, and we then cross-referenced the three data subsets. A total of 26 genes were similarly differentially expressed in all three mutant backgrounds. These included the bHLH transcription factor *FBI1/HFR1/REP1/RSF1* and the putative phytochrome kinase substrate At1g18810, both of which are involved in phytochrome signalling (Fairchild *et al.*, 2000; Schepens *et al.*, 2008). The results showed an extensive overlap in the differential transcriptome of the *siz1* and *spf1/2 siz1* mutants (Fig. 9D). When we compared expression values of *spf1/2 siz1* directly to *siz1*, only 10 genes were down-regulated and six were up-regulated, indicating that their transcriptome virtually matched (Fig. 9E). This transcriptomic data reinforced the notion that SIZ1 was upstream of, and epistatic to, SPF1/2.

## Discussion

Sumoylation is essential for eukaryotic organisms, mainly because it regulates the activity of vital proteins. It is therefore crucial that SUMO homeostasis is tightly controlled, and in recent years some studies have shed light on SUMO protease activities and their essential roles in many aspects of cellular homeostasis (reviewed by Hickey *et al.*, 2012). In plant genomes, as in other organisms, SUMO proteases seem to be more abundant in number than the E1/E2/E3 components of the conjugation machinery, making them prime candidates for the regulation of SUMO conjugation/deconjugation homeostasis. In the present study, we performed a functional characterization of SPF1 and SPF2, two ULP2s that form a separate phylogenetic subgroup within Arabidopsis ULPs. Our results support a conserved evolutionary role for both proteins in plant growth and development.

Previous phylogenetic studies singled out SPF1 and SPF2 as homologs of yeast Ulp2 and mammalian SENP6/7, making them natural candidates for poly-SUMO chain editing proteases in Arabidopsis (Hickey *et al.*, 2012). Here, we report a more thorough phylogenetic and comparative genomics approach that suggests the presence of three ULP2 classes within plant genomes. These display a topological pattern of specific loops within the catalytic domain that separate them from plant ULP1 SUMO proteases (Fig. 2B). In humans, it has been shown that the catalytic domains of the ULP2s SENP6 and SENP7 create loops for SUMO recognition (Lima and Reverter, 2008; Alegre and Reverter, 2011). For example, SENP6/7 loop 1 is essential for activity and SUMO isoform discrimination, but it is not conserved either in yeast or plant ULP2s, highlighting the caveats that must be placed on functional inference based on ULP homology. An interesting characteristic that is intrinsic to the *SPF-type* of plant ULP2s is that the catalytic domain is located in the middle of the protein (Fig. 2A), a feature shared with yeast and algae Ulp2 paralogs, suggesting that this may be the most ancestral group, as opposed to the *OTS*-type of ULP2 proteases. With regards to the function of the N- and C-terminal ends, the model proposed for yeast ULP2 is that the N-terminal domain acts mainly in nuclear targeting (Kroetz *et al.*, 2009), whereas the C-terminal end contains motifs for PTMs such as phosphorylation (Baldwin *et al.*, 2009). In agreement with this, the Arabidopsis SPF1 C-terminal end was previously identified as being a phosphorylation target (PhosPhAt database; Durek *et al.*, 2010). It is important to note that other ULP2-like proteases have previously been proposed by Kurepa *et al.* (2003) and Lois (2010). However, these putative ULP-like genes are part of transposon elements (Hoen *et al.*, 2006) and were designated *Kaonashi ULP-like* (*KIU*) sequences. Although they potentially have catalytically functional domains, their SUMO protease activities have never been studied. Nevertheless, *KIU*s also belong to a phylogenetically distant branch from the remaining ULP family members and are strongly silenced (Hoen *et al.*, 2006), suggesting a minor contribution to SUMO regulation if it is the case that they do function as SUMO proteases.

SUMO proteases can have a dual function as both maturases of the pre-SUMO peptide or as isopetidases that remove SUMO conjugates from targets, and it is important to establish the individual contribution of the different ULPs to each biochemical role. Loss of SPF1/2 function resulted in the constitutive accumulation of high molecular weight SUMO conjugates (Fig. 4), implicating them as SUMO isopeptidases. This is consistent with their phylogenetic proximity with yeast and human ULP2 proteins, both of which display major isopeptidase activity (Lima and Reverter, 2008; Eckhoff and Dohmen, 2015). Here, we further demonstrated that SPF2 was capable of complementing *ulp2∆* but not *ulp1-ts*, placing this plant protease as a functional homolog of the yeast Ulp2. The observed dominant negative effect of SPF1 on *ulp2∆* also suggested a functional correlation with its yeast ortholog, in which the existing topological differences (Fig. 2) may accommodate the observed phenotype. SPF1 and SPF2 both displayed reactivity of their catalytic domain with human HA-SUMO-VME probes, albeit with separate affinities for different SUMO isoforms (Fig. 3B). Our data, combined with recent studies demonstrating endopeptidase activity of SPF1 and SPF2 (Kong *et al.*, 2017; Liu *et al.*, 2017*a*), make a definitive case for SPF1/2 functioning as SUMO proteases.

*In planta*, SPF1 and SPF2 loss-of-function mutants coincided in a series of developmental defects. Several of our results supported the existence of unequal redundancy, tending towards SPF1 as being more important: (1) *SPF1* seemed to be much more expressed than *SPF2*, as shown by semi-quantitative RT-PCR (Supplementary Fig. S3) and by publicly available transcriptomic data (Supplementary Fig. S6); (2) compared to *spf2*, *spf1* mutant alleles displayed more prominent phenotypes in leaf morphology, flowering time, pigment accumulation, and increased SUMO conjugates (Figs 4, 6, 7; Supplementary Fig. S5); (3) several plant genomes display a single-plant SPF1/2 subgroup member (e.g. *Selaginella moellendorffii*, *Oryza sativa*, and *Amborella trichopoda*), and the Arabidopsis SPF1/SPF2 duplication seems to map to a dicot-specific event. Previous functional reports also support this claim (Kong *et al.*, 2017; Liu *et al.*, 2017*a*).

SPF1/2 control a series of development features, making them interesting candidate genes for crop improvement. The *spf1/2* mutant phenotypes included (1) late flowering, indicative of a delay in development; (2) altered leaf morphology; and (3) severely impaired seed production (Fig. 6). However, seeds were also bigger, which may provide an interesting potential for increasing seed size in crop species (Fig. 6L–N). We have shown that SPF1/2 controls several genes involved in secondary metabolism (Fig. 8A, B; Table 1), which may explain the observed developmental defects. For instance, genes involved in glucosinolates and lignin deposition, such as *Ferulic acid 5-hydroxylase* (*F5H*), were down-regulated in *spf1/2*, suggesting that SPF1 and SPF2 act as positive regulators of lignin deposition. Down-regulation of lignin biosynthesis may cause net flux changes through the phenylpropanoid metabolism that could explain why *spf1/2* displayed increased anthocyanin content. In support of this, the metabolic interaction between lignin and anthocyanin biosynthesis has been previously reported (Ring *et al.*, 2013). The observed differences in leaf morphology displayed by both plate-grown and adult *spf1/2* mutants may have reflected changes in either life cycle or cell expansion. Both factors have been associated with SUMO pathway mutants (Murtas *et al.*, 2003; Miura *et al.*, 2010), and both factors contribute to the multiple and complex regulatory modules regulating leaf morphology (Gonzalez *et al.*, 2010). Indeed, several components of the cell wall remodelling apparatus were affected in *spf1/2*, including members of the xyloglucan endotransglucosylase/hydrolase (XTH) family such as *XTH31*, which has previously been observed to be down-regulated in *siz1* (Miura *et al.*, 2010) and was over-expressed in *spf1/2* (Fig. 8C). Most significantly, we have found substantial evidence that many phenotypes displayed by *spf1/2* oppose those of *siz1*, including SUMO-conjugate accumulation, late flowering, higher pigment contents, and reduced accumulation of reactive oxygen species (P.H. Castro *et al*., unpublished results). Here, the *spf1/2 siz1* triple-mutant morphologically resembled the *siz1* single-mutant, suggesting that SPF1/2 are epistatic to SIZ1.

**Table 1.** Genes constitutively deregulated in *spf1/2* compared to the wild-type Col-0

AGI ID	Gene name	Log_2_ ratio	*P*-value	Description
**Hormone metabolism**				
Auxin				
At1g77690	*LAX3*	–0.65	2.41 × 10^–4^	Auxin influx carrier
At5g35735		0.58	9.44 × 10^–3^	Auxin-responsive
At1g56150		0.59	6.19 × 10^–3^	SAUR-like auxin-responsive
At4g14560	*AXR5, IAA1*	0.88	2.49 × 10^–10^	Aux/IAA protein
At5g18060	*SAUR23*	0.96	<0.01 × 10^–11^	SAUR-like auxin-responsive
Brassinosteroid				
At3g30180	*BR6OX2*, *CYP85A2*	1.30	<0.01 × 10^–11^	Brassinosteroid-6-oxidase
Cytokinin				
At1g22400	*UGT85A1*	0.64	5.00 × 10^–4^	UDP-glycosyltransferase
Gibberellin				
At2g14900		0.65	2.58 × 10^–4^	Gibberellin-regulated
At5g25900	*KO1*, *CYP701A3*, *GA3*	0.71	1.45 × 10^–5^	Kaurene oxidase
Jasmonate				
At1g52070		0.61	2.07 × 10^–3^	Mannose-binding lectin
At5g42650	*AOS*, *CYP74A*, *DDE2*	0.81	2.26 × 10^–8^	Allene oxide synthase
At1g52100		1.09	<0.01 × 10^–11^	Mannose-binding lectin
Salicylic acid				
At5g38020		0.70	2.23 × 10^–5^	SAM-Mtases
At5g37990		0.82	1.61 × 10^–8^	SAM-Mtases
**Secondary metabolism**				
Phenylpropanoids (lignin biosynthesis)				
At4g37980	*CAD7*, *ELI3*	–1.13	<0.01 × 10^–11^	Cinnamyl alcohol dehydrogenase
At5g66690	*UGT72E2*	–0.81	3.20 × 10^–8^	UDP-glycosyltransferase
At4g39330	*CAD9*	–0.66	1.29 × 10^–4^	Cinnamyl alcohol dehydrogenase
At4g36220	*CYP84A1*, *FAH1*, *F5H*	–0.56	2.57 × 10^–2^	Ferulic acid 5-hydroxylase
Lipids				
At1g06080	*ADS1*	–1.51	<0.01 × 10^–11^	Acyl-lipid/acyl-CoA desaturase
At5g14180	*MPL1*	–1.50	<0.01 × 10^–11^	*Myzus persicae*-induced lipase
At5g04530	*KCS19*	–1.02	<0.01 × 10^–11^	3-ketoacyl-CoA synthase
At1g06350		–0.91	4.48 × 10^–11^	Fatty acid desaturase
At3g08770	*LTP6*	–0.91	4.48 × 10^–11^	Lipid transfer protein
At4g34250	*KCS16*	–0.62	1.47 × 10^–3^	3-ketoacyl-CoA synthase
At3g11670	*DGD1*	–0.60	2.80 × 10^–3^	UDP-glycosyltransferase
At4g38690		–0.56	1.92 × 10^–2^	PLC-like phosphodiesterase
Glucosinolates				
At3g14210	*ESM1*	–1.72	<0.01 × 10^–11^	Epithiospecifier modifier
At4g13770	*CYP83A1*, *REF2*	–0.74	2.35 × 10^–6^	Cytochrome P450
At2g43100	*LEUD1*, *IPMI2*	–0.68	5.43 × 10^–5^	Isopropylmalate isomerase
At5g23010	*IMS3*, *MAM1*	–0.64	5.52 × 10^–4^	Methylthioalkylmalate synthase
At1g07640	*OBP2*	–0.60	3.01 × 10^–3^	DOF transcription factor
At3g44320	*NIT3*	0.75	1.26 × 10^–6^	Nitrilase
At1g54010	*GLL22*	0.90	6.97 × 10^–11^	GDSL-like lipase/acylhydrolase
Cell wall				
At5g65730	*XTH6*	–1.61	<0.01 × 10^–11^	XTH
At1g67750		–0.66	1.31 × 10^–4^	Pectate lyase
At5g47500	*PME5*	–0.63	8.57 × 10^–4^	Pectin methylesterase
At4g28250	*EXPB3*	–0.59	6.49 × 10^–3^	Beta-expansin
At3g23730	*XTH16*	–0.59	6.24 × 10^–3^	XTH
At1g20190	*EXPA11*	0.57	1.08 × 10^–2^	Alpha-expansin
At1g55850	*CSLE1*	0.57	1.49 × 10^–2^	Cellulose synthase/transferase
At3g29810	*COBL2*	0.59	4.76 × 10^–3^	COBRA-like protein precursor
At2g06850	*XTH4*, *EXGT-A1*, *EXT*	0.63	6.61 × 10^–4^	XTH
At3g28180	*CSLC4*	0.78	1.94 × 10^–7^	Cellulose synthase/transferase
At4g30290	*XTH19*	0.88	2.09 × 10^–10^	XTH
At5g33290	*XGD1*	0.95	<0.01 × 10^–11^	Xylogalacturonan xylosyltransferase
At3g44990	*XTH31*, *XTR8*	1.29	<0.01 × 10^–11^	XTH
**Other**				
At2g45660	*SOC1*, *AGL20*	–0.83	6.01 × 10^–9^	AGAMOUS-like transcription factor
At1g77760	*NIA1*, *GNR1*, *NR1*	–0.83	7.34 × 10^–9^	Nitrate reductase
At4g21680	*NRT1.8*	0.61	1.81 × 10^–3^	Nitrate transporter
At5g50200	*NRT3.1*, *WR3*	0.62	1.11 × 10^–3^	Nitrate transporter

XTH, Xyloglucan endotransglucosylase/hydrolase; SAM-Mtases, S-adenosyl-L-methionine-dependent methyltransferase.

The categories were chosen taking in consideration the enrichment of gene ontology (GO) terms, and the list was gathered using Classification SuperViewer (Toufighi *et al.*, 2005) and The Arabidopsis Information Resource (TAIR) (Lamesch *et al.*, 2010).

Both mammalian SENP and yeast ULP vary in their subnuclear localization (reviewed by Wilkinson and Henley, 2010) and contribute differently to SUMO dynamics within the nucleus. In Arabidopsis, ULPs display a variety of sub-cellular localizations: ESD4 in the nuclear envelope, OTS2 in speckle-like bodies of the nucleoplasm, OTS1 in the nucleoplasm, and ELS1 in the cytoplasm and endomembranes (Murtas *et al.*, 2003; Conti *et al.*, 2008; Hermkes *et al.*, 2011). Recently, SPF1 and SPF2 were both localized in the nucleus (Kong *et al.*, 2017; Liu *et al.*, 2017*a*). In addition, we observed that SPF1 and SPF2 were both located in the nucleoplasm and in nuclear bodies (Fig. 5). In accordance with this, plant SUMO conjugates are mainly nuclear-targeted proteins and ULPs contribute to the regulation of nuclear SUMO dynamics (Saracco *et al.*, 2007; Elrouby and Coupland, 2010; Miller *et al.*, 2010). Among SUMO targets are transcription factors, co-repressor complexes, histones, mRNA biogenesis proteins, and many other components associated with nuclear processes (Mazur and van den Burg, 2012). In addition to previous reports that SIZ1 and OTS1/2 significantly influence the plant transcriptome (Castro *et al.*, 2016; Catala *et al.*, 2007), SPF1/2 were also involved in transcription regulation, and seemed to mainly influence secondary metabolism, N-assimilation, and flowering time. Some of the DEGs that we found such as *NIA1*, *SOC1*, and *XTH31* (Fig. 8; Table 1) have previously been associated with SIZ1 regulation but with the opposite behaviour. As previously noted, the *spf1/2 siz1* triple-mutant phenotypically resembled *siz1* and, accordingly, the transcriptional profile of *spf1/2 siz1* was superimposed on that of *siz1* but not *spf1/2*. Taken together, SPF1/2 function seemed to take place downstream of SIZ1. The simplest model is that targets of SIZ1-dependent sumoylation are subjected to SPF1/2 desumoylation. Most bona fide candidates include transcription factors such as PHR1, ICE1, ABI5, HSFA2, and MYB30 (Miura *et al.*, 2005, 2007*b*, 2009; Cohen-Peer *et al.*, 2010; Zheng *et al.*, 2012). *Cis*-element enrichment analysis also highlighted MYC2 as a potential target for SPF1/2 regulation (Supplementary Table S4), and in support of this MYC2 has previously been shown to be sumoylated *in vitro* (Elrouby and Coupland, 2010).

Sumoylation of target proteins is largely under the control of SIZ1 E3 ligase activity (Miura *et al.*, 2005; Catala *et al.*, 2007). Although many SUMO machinery components are sumoylated under normal conditions, SIZ1 is the only heavily sumoylated protein under stress conditions (e.g. HS, ethanol, and H_2_O_2_) (Miller *et al.*, 2013). One possibility is that SIZ1 may be one of the major targets of SPF1/2. In accordance with this hypothesis, yeast Siz1 and Siz2 are high-copy suppressors of *ulp2*Δ phenotypes, suggesting that the requirement for yeast Ulp2 is bypassed by SIZ1 overexpression (Strunnikov *et al.*, 2001; Hannich *et al.*, 2005). However, plants might display higher complexity, since *spf1/2* and *siz1* revealed opposing phenotypes in our current study and their transcriptomes were not significantly co- or inversely expressed (Fig. 9E).

An often-neglected aspect to consider when addressing Arabidopsis ULPs is a possible functional redundancy between different ULP subgroup members. For example, *esd4* and *ots1/2* mutants have been shown to accumulate high molecular weight SUMO conjugates under non-stress conditions (Murtas *et al.*, 2003; Xu *et al.*, 2007; Conti *et al.*, 2008; Castro *et al.*, 2016) and ESD4, ELS1, OTS1, and OTS2 have shown SUMO1/2 isopeptidase activity *in vitro* (Chosed *et al.*, 2006; Colby *et al.*, 2006; Conti *et al.*, 2008; Hermkes *et al.*, 2011). On the other hand, we have previously reported that the triple-mutant *ots1/2 siz1* showed accumulative defects, which partially place OTS1/2 and SIZ1 in different pathways (Castro *et al.*, 2016). The *esd4 siz1* mutant, like *spf1/2 siz1*, resembles *siz1* (P.H. Castro *et al*., unpublished results), but SIZ1 and ESD4 are also likely to function in different pathways since the *siz1* pleiotropic phenotype is largely reverted in the *NahG* background (expressing a bacterial SA hydroxylase that hydrolyses SA), while *esd4* is not (Hermkes *et al.*, 2011). However, more recently Villajuana-Bonequi *et al.* (2014) reported that a mutation in the *ICS1/SID2* gene, a key enzyme in SA biosynthesis, is able to partially suppress *esd4* developmental defects, suggesting that ESD4 and SIZ1 may overlap in some functions. Discriminating desumoylation targets for each ULP will be an important step towards dissecting the circuitry of regulation via SUMO removal, and ultimately identifying the origin of specificity within the sumoylation pathway. This goal can be achieved by combining mutant backgrounds of ULPs with previously demonstrated high-throughput strategies for identifying sumoylomes (Miller *et al.*, 2010).

## Supplementary data

Supplementary data are available at *JXB* online.

Fig. S1. Protein sequence alignment of the catalytic domain of SPF1/2 subgroup members.

Fig. S2. Purification elution of recombinant proteins with the SPF2 and SPP1 catalytic domains with an N-terminus GST-tag.

Fig. S3. Schematic representation of Arabidopsis T-DNA insertion mutants for *SPF2* and *SPF1* and semi-quantitative RT-PCR.

Fig. S4. Plant and silique size of the wild-type Col-0 and *spf1/2* mutant.

Fig. S5. Morphology of 1-month-old plants of the *SPF2* and *SPF1* second-allele T-DNA mutant.

Fig. S6. *In silico* analysis of *SPF2* and *SPF1* expression patterns.

Table S1. List of primers used for genotyping Arabidopsis T-DNA insertion lines.

Table S2. List of primers used in semi-quantitative and quantitative RT-PCR.

Table S3. List of primers used for plasmid constructs.

Table S4. *Cis*-elements over-represented in the promoter region of differentially expressed genes in *spf1/2*.

Supplementary Material
