# Supplementary material for: Arabidopsis thaliana SPF1 and SPF2 are nuclear-located ULP2-like SUMO proteases that act downstream of SIZ1 in plant development
Source: J Exp Bot. 2018 Jul 25;69(19):4633–49. doi: 10.1093/jxb/ery265 (PMC6117582; doi:10.1093/jxb/ery265)
Supplement: Supplementary Material [file ery265_suppl_supplementary_material.pdf]

**Title**

***Arabidopsis thaliana*** **SPF1 and SPF2 are nuclear-located ULP2-like SUMO proteases that act downstream of SIZ1 in plant development**

**Authors**

Pedro Humberto Castro

Miguel Ângelo Santos

Sara Freitas

Pepe Cana-Quijada

Tiago Lourenço

Mafalda A. A. Rodrigues

Fátima Fonseca

Javier Ruiz-Albert

Jorge E. Azevedo

Rui Manuel Tavares

Araceli G. Castillo

Eduardo R. Bejarano

Herlânder Azevedo

**Supplementary data**

**Fig. S1.** Protein sequence alignment of the catalytic domain of SPF1/2 subgroup members.

**Fig. S2.** Purification elution of recombinant proteins with the SPF2 and SPP1 catalytic domains with an N-terminus GST-tag.

**Fig. S3.** Schematic representation of Arabidopsis T-DNA insertion mutants for *SPF2* and *SPF1* and semi-quantitative RT-PCR.

**Fig. S4.** Plant and silique size of the wild-type Col-0 and *spf1/2* mutant.

**Fig. S5.** Morphology of 1-month-old plants of the *SPF2* and *SPF1* second allele T-DNA mutant.

**Fig. S6.** *In silico* analysis of *SPF2* and *SPF1* expression patterns.

**Table S1.** List of primers used for genotyping Arabidopsis T-DNA insertion lines.

**Table S2.** List of primers used in semi-quantitative and quantitative RT-PCR.

**Table S3.** List of primers used for plasmid constructs.

**Table S4.** *Cis*-elements over-represented in the promoter region of differentially expressed genes in *spf1/2*.

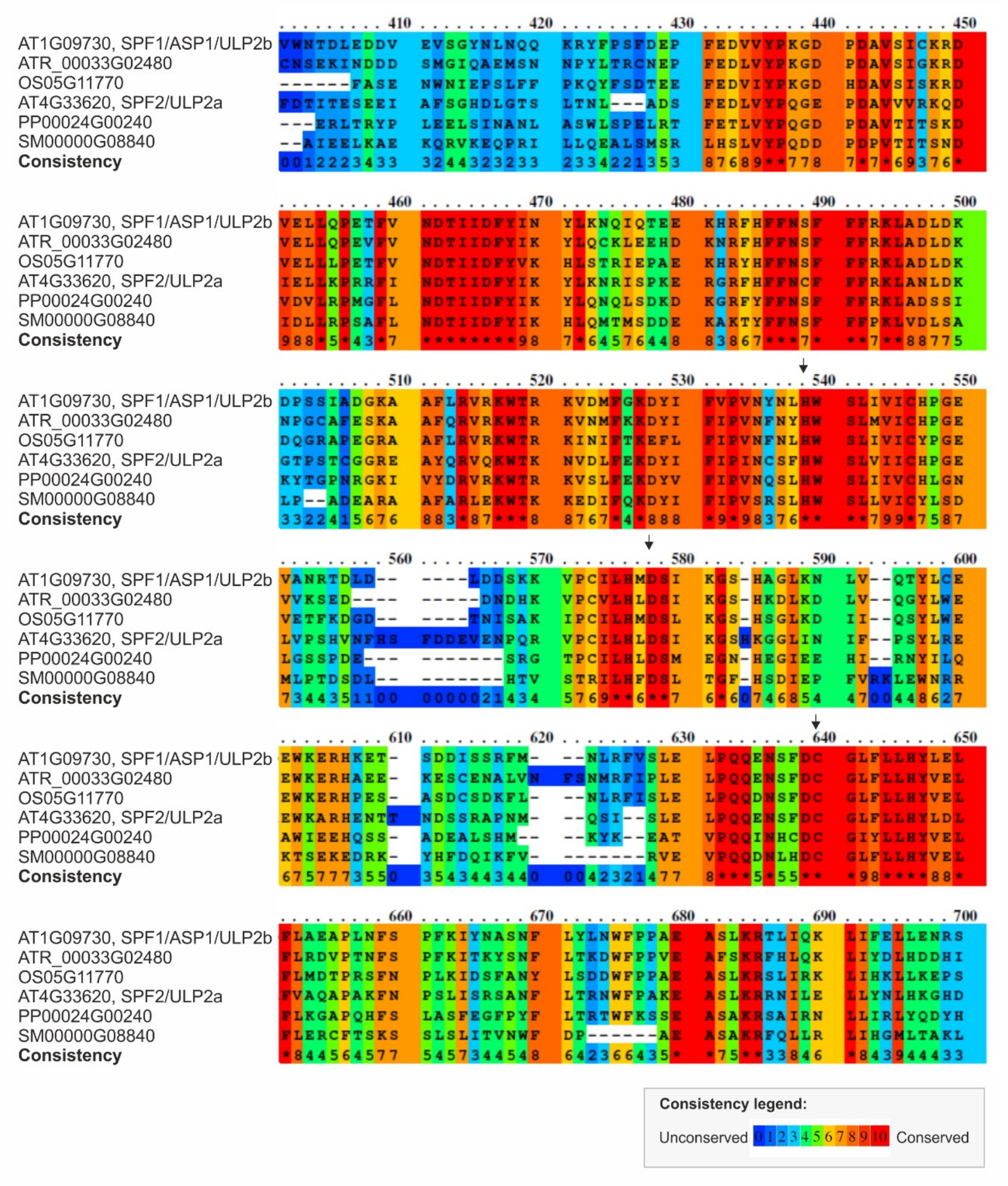


**Fig. S1.** Protein sequence alignment of the catalytic domain of SPF1/2 subgroup members. Protein sequences were retrieved from *Arabidopsis thaliana*, *Physcomitrella patens*, *Selaginella moellendorffii*, *Amborella trichopoda*, and *Oryza sativa* ssp. Japonica genomes, presenting the three conserved catalytic residues (arrows). Consistency between sequences (colour gradient) indicates the level of conservation of each residue. Protein sequence alignment was performed using PRALINE (http://www.ibi.vu.nl/programs/pralinewww/).

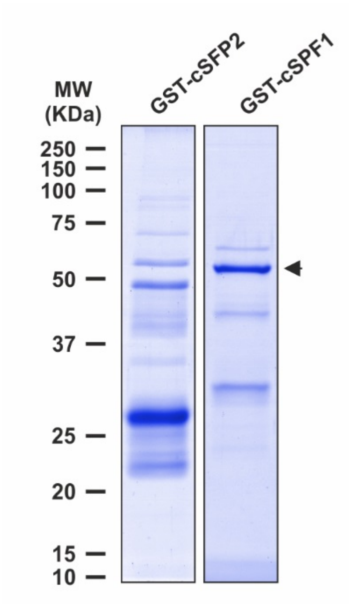


**Fig. S2.** Purification elution of recombinant proteins with the SPF2 and SPF1 catalytic domains with an N-terminus GST-tag. A protein elution aliquot ran in 10% SDS-PAGE and was stained with *Xpert Safe Protein Stain* (Grisp). Arrow indicates approximately the expected molecular weight for GST-SPFs recombinant proteins.

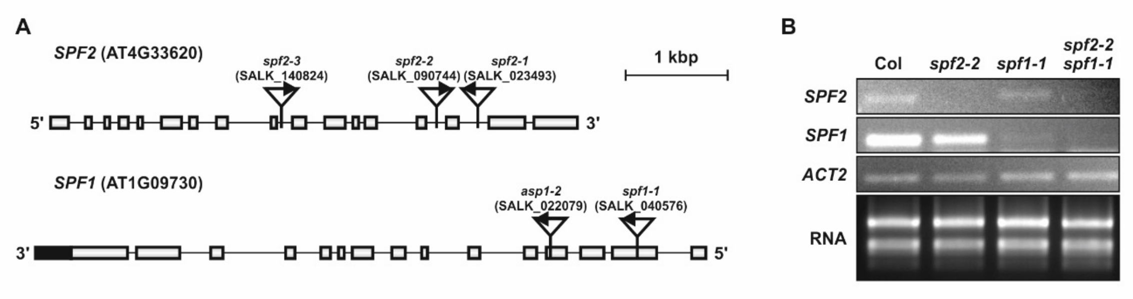


**Fig. S3.** Schematic representation of Arabidopsis T-DNA insertion mutants for *SPF2* and *SPF1* and semi-quantitative RT-PCR. (**A**) Schematic representation of *SPF2* and *SPF1* displaying exons (grey boxes), introns (thin lines), and UTRs (black boxes). The site and orientation of T-DNA insertions are represented by triangles with the respective SALK line code. Scale bar indicates 1 Kbp. (**B**) Semi-quantitative RT-PCR for wild-type (Col), *spf2-2* (*spf2*), *spf1-1* (*spf1*) and *spf2-2 spf1-1* (*spf1/2*). *ACT2* was used as a loading control and the total extracted RNA, used as template for reverse transcription, served as a quality control.

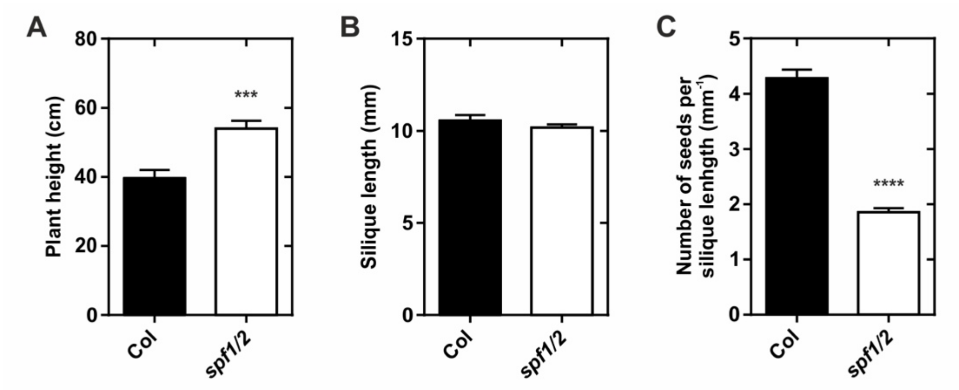


**Fig. S4.** Plant and silique size of the wild-type Col-0 and *spf1/2* mutant. Plant size at the end of life cycle (**A**), silique size (**B**) and number of seeds normalized by silique size (**C**). Error bars represent standard error of the means (SEM), n > 10 (A), and n = 9 (B, C). Asterisks indicate statistically significant differences with respect to the wild-type (unpaired t test; ***, P < 0.001; ****, P < 0.0001).

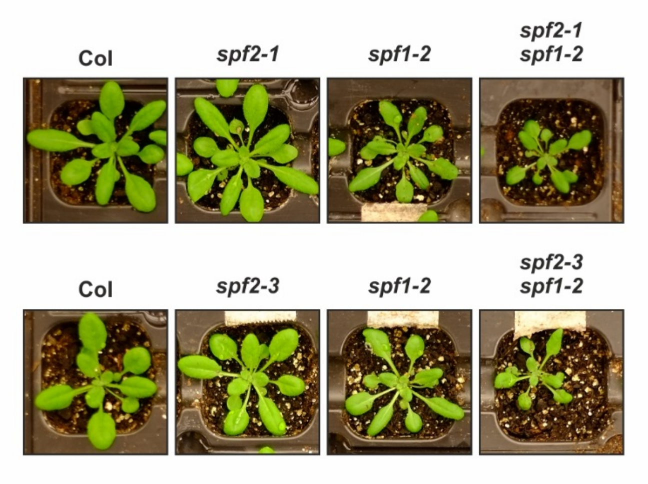


**Fig. S5.** Morphology of 1-month-old plants of the *SPF2* and *SPF1* second allele T-DNA mutants.

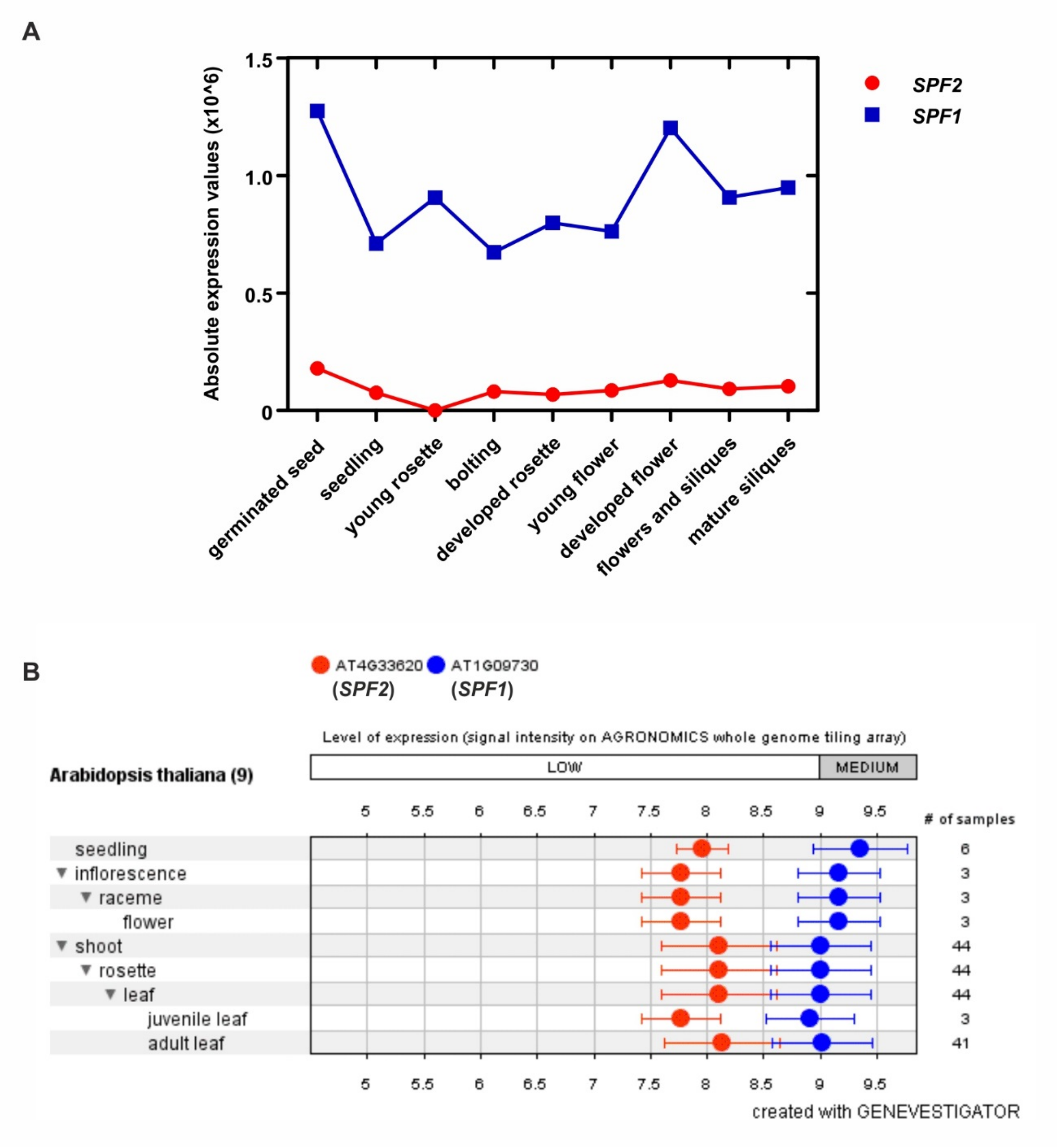


**Fig. S6.** *In silico* analysis of *SPF2* and *SPF1* expression patterns. Expression profile of Arabidopsis *SPF2* (red) and *SPF1* (blue) during development (**A**) and in different plant organs (**B**). Analysis was conducted in Genevestigator (genevestigator.com).

**Table S1.** List of primers used for genotyping Arabidopsis T-DNA insertion lines.

| **Primer name** | **Primer sequence (5' to 3')** | **Description** |
|---|---|---|
| SPF2-2 LP | ACCCACAAAGGGTTCCCTGCAT | Genotyping of *spf2-2* (SALK_090744) |
| SPF2-2 RP | TCTCTTGCTGCGGGAGCTGA | |
| SPF2-3 LP | CCAACTTTACAAGCTGCTTCG | Genotyping of *spf2-3* (SALK_140824) |
| SPF2-3 RP | TGCTGACAGGAATTGATCTCC | |
| SPF2-1 LP | AATCAGTTTTGGTCGGTAGGC | Genotyping of *spf2-1* (SALK_023493) |
| SPF2-1 RP | AAAGCATGCATCCCTCTTTTC | |
| SPF1-1 RP | TCCGCCTAGCTGGCAAGAGA | Genotyping of *spf1-1* (SALK_040576) |
| SPF1-1 LP | CCGGTTCTGCAACGCCAACG | |
| SPF1-2 RP | GTCAACGCCAGCTAAACTCAC | Genotyping of *spf1-2* (SALK_022079) |
| SPF1-2 LP | CCAAAATCGAATGAAAGCATG | |
| SIZ1-2 RP | CACGACAGATGAAGCATTGTG | Genotyping of *siz1-2* (SALK_065397) |
| SIZ1-2 LP | GAGCTGAAGCATCTGGTTTTG | |
| LBb1.3 | ATTTTGCCGATTTCGGAAC | Left border primer for genotyping SALK T-DNA insertion lines |

**Table S2**. List of primers used in semi-quantitative and quantitative RT-PCR.

| Gene (AGI code) | Primer name | Primer sequence (5' to 3') | Tm | GC (%) | Product size (bp) |
|---|---|---|---|---|---|
| *ACT2* | ActinF | CTAAGCTCTCAAGATCAAAGGCTTA | 52.7 | 40.0 | 211 |
| (At3g18780) | ActinR | ACTAAAACGCAAAACGAAAGCGGTT | 57.2 | 40.0 | |
| *SPF2* | SPF2 RT F | CTTTTGACTGTGGCCTCTT | 49.6 | 47.4 | 183 |
| (At4g33620) | SPF2 RT R | CTTTGTGGAGGTTGTAAAGC | 49.1 | 45.0 | |
| *SPF1* | SPF1 RT F | GGAAGAAGAAATGGAAGGTC | 47.8 | 45.0 | 152 |
| (At1g09730) | SPF1 RT R | CTAAATGGTCAGTGGTTTCC | 48.2 | 45.0 | |
| *CAD7* | CAD7 RT F1 | TTTCCTCTCATCTTTGGGCG | 57.9 | 50.0 | 144 |
| (At4g37980) | CAD7 RT R1 | GGCGGTGTTGACATAATCCG | 59.3 | 55.0 | |
| *KNAT1* | KNAT1 RT F1 | AGTGGCCATATCCTTCTGAGTC | 59.3 | 50.0 | 181 |
| (At4g08150) | KNAT1 RT R1 | TCCATGTACAGAGCTGCGTG | 60.1 | 55.0 | |
| *NIA1* | NIA1 RT F1 | CCACCAGGAGAAACCGAACA | 59.9 | 55.0 | 167 |
| (At1g77760) | NIA1 RT R1 | TCATCCCCATGAGGTTCCAG | 58.9 | 55.0 | |
| *PER1* | PER1 RT F1 | TTCGCCAATTCTTGGACCGT | 60.2 | 50.0 | 199 |
| (At1g48130) | PER1 RT R1 | CCTTGCTTCCGTGATTAAAGGC | 60.2 | 50.0 | |
| *SOC1* | SOC1 RT F1 | GAGCAGCTCAAGCAAAAGGAGA | 61.1 | 50.0 | 137 |
| (At2g45660) | SOC1 RT R1 | GGGCTACTCTCTTCATCACCTC | 59.6 | 54.6 | |
| *XTH6* | XTH6 RT Fw1 | TGATCAGAGCACTGGATGTGG | 59.8 | 52.4 | 153 |
| (At5g65730) | XTH6 RT Rv1 | TCTAGCTCGTCTCTCACCGT | 59.8 | 55.0 | |
| *XTH31* | XTH31 RT F1 | TCCACTGGGAGTGGGTTCA | 60.1 | 57.9 | 191 |
| (At3g44990) | XTH31 RT R1 | GAATAAGGCTTCCCTGGCGT | 60.1 | 55.0 | |

*Tm* - Melting temperature; *bp* - Base pair

**Table S3**. List of primers used for plasmid constructs.

| Primer name | Primer sequence (5' to 3') | Description |
|---|---|---|
| SPF2 pENTR Fw1 | TTGCGGCCGCCATGACTCTCCGGTCAGTTCAATCC | Cloning into *pENTR*; RS: *Not*I |
| SPF2 pENTR Rv1 | TTGGCGCGCCCAGTTTTTGGCTTGGCCATCACATT | Cloning into *pENTR*; RS: *Asc*I |
| SPF1 pENTR Fw1 | AAGCGGCCGCCATGAAGAAAAACTTTGAAGTATTCG | Cloning into *pENTR*; RS: *Not*I |
| SPF1 pENTR Rv1 | AAGGCGCGCCCTTTCTCCATCTCCTCAGCTTCGCC | Cloning into *pENTR*; RS: *Asc*I |
| SPF2 pCM190 Fw1 | AGCTTTgtttaaacATGACTCTCCGGTCAGTTC | Cloning into *pCM190*; RS: *Pme*I |
| SPF2 pCM190 Rv1 | ATAGTTTAGCGGCCGCTCAAGTTTTTGGCTTGGCC | Cloning into *pCM190*; RS; *Not*I |
| cSPF2 pGEX5 Fw1 | TTGGATCCAAGATCTAGTTTACCCTCAAGGAG | Cloning into *pGEX-5X-1*; RS: *Bam*HI |
| cSPF2 pGEX5 Rv1 | AATAGCGGCCGCTTAACCTTTGTGGAGGTTGTAA | Cloning into *pGEX-5X-1*; RS: *Not*I |
| cSPF1 pGEX5 Fw1 | AAGAATTCGAGGATGTTGTCTATCCAAAGGGTG | Cloning into *pGEX-5X-1*; RS: *Eco*RI |
| cSPF1 pGEX5 Rv1 | AACTGCGGCCGCTTAGTTTTCAAGGAGTTCAAAT | Cloning into *pGEX-5X-1*; RS: *Not*I |

*RS* – Restriction site

**Table S4.** *Cis*-elements over-represented in the promoter region of differentially expressed genes in *spf1/2*. The DEGs were submitted to Athena analysis (O'Connor *et al*., 2005; DOI: 10.1093/bioinformatics/bti714), to scan for binding site enrichment.

| ***Cis*-element name** | ***Cis*-element sequence*** | **Nr. of genes** | **Predicted in the genome** | **Found in the genes** | ***p*-value** | **Corresponding TFs** |
|---|---|---|---|---|---|---|
| ***Down-regulated*** | | | | | | |
| ***AtMYC2 BS in RD22*** | CACATG | 61 | 35% | 53% | < 10e-6 | MYC2 |
| ***MYCATERD1*** | CATGTG | 61 | 35% | 53% | < 10e-6 | MYC2 |
| ***Up-regulated*** | | | | | | |
| ***AtMYC2 BS in RD22*** | CACATG | 47 | 35% | 47% | < 10e-3 | MYC2 |
| ***MYCATERD1*** | CATGTG | 47 | 35% | 47% | < 10e-3 | MYC2 |
| ***CARGCW8GAT*** | CWWWWWWWWG | 70 | 59% | 70% | < 10e-3 | AGL15 |
| ***TATA-box Motif*** | TATAAA | 91 | 91% | 82% | < 10e-4 | |

** R (A/G), M (A/C), W (A/T), K (G/T), B (C/G/T), N (A/C/G/T)*
